# Ginger Constituent 6-Shogaol Inhibits Inflammation- and Angiogenesis-Related Cell Functions in Primary Human Endothelial Cells

**DOI:** 10.3389/fphar.2022.844767

**Published:** 2022-02-25

**Authors:** Iris Bischoff-Kont, Tobias Primke, Lea S. Niebergall, Thomas Zech, Robert Fürst

**Affiliations:** ^1^ Institute of Pharmaceutical Biology, Goethe University Frankfurt, Frankfurt, Germany; ^2^ LOEWE Center for Translational Biodiversity Genomics (LOEWE-TBG), Frankfurt, Germany

**Keywords:** *Zingiber officinale* Roscoe, 6-shogaol, endothelial cells, inflammation, angiogenesis-related cell functions

## Abstract

Rhizomes from *Zingiber officinale* Roscoe are traditionally used for the treatment of a plethora of pathophysiological conditions such as diarrhea, nausea, or rheumatoid arthritis. While 6-gingerol is the pungent principle in fresh ginger, in dried rhizomes, 6-gingerol is dehydrated to 6-shogaol. 6-Shogaol has been demonstrated to exhibit anticancer, antioxidative, and anti-inflammatory actions more effectively than 6-gingerol due to the presence of an electrophilic Michael acceptor moiety. *In vitro*, 6-shogaol exhibits anti-inflammatory actions in a variety of cell types, including leukocytes. Our study focused on the effects of 6-shogaol on activated endothelial cells. We found that 6-shogaol significantly reduced the adhesion of leukocytes onto lipopolysaccharide (LPS)-activated human umbilical vein endothelial cells (HUVECs), resulting in a significantly reduced transmigration of THP-1 cells through an endothelial cell monolayer. Analyzing the mediators of endothelial cell–leukocyte interactions, we found that 30 µM of 6-shogaol blocked the LPS-triggered mRNA and protein expression of cell adhesion molecules. In concert with this, our study demonstrates that the LPS-induced nuclear factor κB (NFκB) promoter activity was significantly reduced upon treatment with 6-shogaol. Interestingly, the nuclear translocation of p65 was slightly decreased, and protein levels of the LPS receptor Toll-like receptor 4 remained unimpaired. Analyzing the impact of 6-shogaol on angiogenesis-related cell functions *in vitro*, we found that 6-shogaol attenuated the proliferation as well as the directed and undirected migration of HUVECs. Of note, 6-shogaol also strongly reduced the chemotactic migration of endothelial cells in the direction of a serum gradient. Moreover, 30 µM of 6-shogaol blocked the formation of vascular endothelial growth factor (VEGF)-induced endothelial sprouts from HUVEC spheroids and from murine aortic rings. Importantly, this study shows for the first time that 6-shogaol exhibits a vascular-disruptive impact on angiogenic sprouts from murine aortae. Our study demonstrates that the main bioactive ingredient in dried ginger, 6-shogaol, exhibits beneficial characteristics as an inhibitor of inflammation- and angiogenesis-related processes in vascular endothelial cells.

## Introduction

The use of ginger (*Zingiber officinale* Roscoe, Zingiberaceae) for medical purposes in India and China goes back to ancient times (Prasad and Tyagi, 2015). Ginger has been widely used for the treatment of pain, nausea, vomiting (Palatty et al., 2013), rheumatoid arthritis (Al-Nahain et al., 2014), hypertension, kinesalgia, fever, or infectious diseases (Ali et al., 2008) in traditional medicine. According to the Committee on Herbal Medicinal Products (HMPC) of the European Medicines Agency (EMA), powdered ginger rhizomes have the status of “well-established use” for the prevention of nausea and vomiting in motion sickness (European Medicines Agency, 2013). More than 400 ingredients of ginger have been described; among them, the major pungent constituent in fresh ginger is 6-gingerol. During storage, 6-gingerol (Supplementary Figure S1A) is degraded in dried ginger, while the quantity of 6-shogaol (Supplementary Figure S1B) increases due to dehydration processes (Jolad et al., 2005). 6-Shogaol was first identified by Nomura (1918), and since then, numerous preclinical studies have investigated the characteristics and potential effects of 6-shogaol. *In vitro* and *in vivo*, 6-shogaol has been shown to exhibit anticancer, anti-inflammatory, antioxidant, and neuroprotective actions (Suekawa et al., 1984; Prasad and Tyagi, 2015; Nonaka et al., 2019; Ma et al., 2021). Comparative studies demonstrate beneficial effects of 6-shogaol over 6-gingerol with regard to anticancer, antioxidative, and anti-inflammatory effects that might be attributed to the chemical structure of 6-shogaol, containing an α,β-unsaturated carbonyl moiety (Michael acceptor) (Sang et al., 2009; Dugasani et al., 2010; Kou et al., 2018). Anti-inflammatory actions of 6-shogaol have been studied in a variety of preclinical studies. *In vivo*, 6-shogaol successfully reduced the formation of paw edema, leukocyte infiltration into the tissue, or symptoms of arthritis (Levy et al., 2006). By studying the effects on underlying mechanisms, it was demonstrated that 6-shogaol impairs the nuclear factor κB (NFκB) pathway and nuclear factor erythroid 2-related factor 2 (Nrf2)/heme oxygenase 1 (HO-1)-signaling cascade (Han et al., 2019). We recently summarized in a review article the benefits of ginger and, in particular, of its constituent 6-shogaol for the inhibition of inflammation-related processes (Bischoff-Kont and Fürst, 2021).

The vascular endothelium plays a pivotal role in the process of inflammation as it represents a barrier for solutes and immune cells and is crucially involved in the process of angiogenesis. In chronic inflammatory diseases, such as rheumatoid arthritis or psoriasis, leukocytes are constantly infiltrating the underlying tissue through the vascular endothelial barrier resulting in severe damage. Concurrently, vascular endothelial cells, which form the inner lining of blood vessels, are perpetually activated and, moreover, continuously perform angiogenic sprouting, accelerating the leukocyte infiltration (Aguilar-Cazares et al., 2019). Therefore, a strategy addressing both characteristics of endothelial cell activation is desirable: stabilization of the endothelial cell barrier for leukocyte diapedesis and downregulation of angiogenic cell functions. Interestingly, only few studies addressed the potential anti-inflammatory and antiangiogenic effects of 6-shogaol in the vascular endothelium or vascular endothelial cells (Weng et al., 2012). The aim of this study was to demonstrate the effects of 6-shogaol on endothelial cells that have been activated. For inflammatory activation in endothelial cells, we show the effects of 6-shogaol after cytokine- and lipopolysaccharide (LPS)-induced activation and the consequential effects on leukocyte–endothelial cell interactions. Moreover, we demonstrate 6-shogaol-derived effects on key steps of angiogenesis-related cell functions: 1) endothelial cell proliferation, 2) directed migration, and 3) endothelial cell sprouting *in vitro* and *ex vivo.*

## Materials and Methods

### Compounds

6-Shogaol was purchased from PhytoLab GmbH & Co. KG (Vestenbergsgreuth, Germany) and 6-gingerol from MedChemExpress (Monmouth Junction, NJ, United States) and was used in the experiments in different concentrations in a range between 1 and 100 µM. The concentration used in each experiment is stated in the respective figure. Human recombinant interleukin 1β (IL-1β), tumor necrosis factor (TNF), stromal cell-derived factor 1 (SDF-1), vascular endothelial growth factor (VEGF_165_), and murine recombinant VEGF_165_ were obtained from PeproTech (Rocky Hill, NJ, United States). Lipopolysaccharide (LPS) from *Escherichia coli* O127:B8 was from Sigma-Aldrich (St. Louis, MO, United States). The concentrations of 6-shogaol for the treatment of endothelial cells were chosen up to the maximum concentration without inducing cytotoxicity. The concentration of 0.1 up to 150 µM was previously used for other cell types (Bischoff-Kont and Fürst, 2021). For 6-gingerol, the same concentration range was used. Moreover, a study employing HUVECs for the analysis of effects of 6-gingerol on microvessel-like structure formation used concentrations of up to 30 µM (Zhog et al., 2019). The concentration of 10 ng/ml of TNF is an established hyperphysiological concentration that is frequently used in order to obtain a strong inflammatory induction in HUVECs (Stark et al., 2019; Krishnatas et al., 2021). For IL-1β, 5 ng/ml was found to be a reasonable concentration to induce the inflammatory response in endothelial cells and is within the commonly applied concentration range (Kihara et al., 2021). The applied concentration of human VEGF_165_ for the *in vitro* assays resulted in appropriate angiogenic induction and is commonly used for angiogenesis-related experiments in endothelial cells (Liu et al., 2009; Bräutigam et al., 2019; Bischoff-Kont et al., 2021). Murine VEGF_165_ was applied according to the publication of Baker et al. (2011). LPS was applied in order to induce an appropriate inflammatory response in HUVECs according to the concentrations recommended in the literature (Zeuke et al., 2002). The applied concentration of SDF-1 was used to induce the migration of THP-1 cells, and the concentration of 500 ng/ml corresponds to data from the literature (Aiuti et al., 1997).

In this study, established pharmacological positive controls have not been included, since key experiments and the respective actions of active pharmacological compounds that substitute for a positive control have already been published by our group (Bräutigam et al., 2019; Stark et al., 2019; Bischoff-Kont et al., 2021; Krishnatas et al., 2021).

### Cell Culture

HUVECs were isolated according to Jaffe et al. (1973) or obtained from PELOBiotech (Planegg/Martinsried, Germany). For isolation of HUVECs, veins of human umbilical cords were exempted from residual blood clots with sterile phosphate-buffered saline (PBS) before a collagenase A (F. Hoffmann-La Roche, Basel, Switzerland) solution was filled into the vein and incubated for 45 min at 37°C to detach HUVECs. Subsequently, the umbilical vein was washed with medium 199 (PAN-Biotech, Aidenbach, Germany) containing 100 U/ml penicillin, 100 μg/ml streptomycin (PAN-Biotech, Aidenbach, Germany), and 10% fetal calf serum (FCS; Biochrom, Berlin, Germany), and the detached cells were centrifuged for 5 min at 300 g. The pellet was resuspended in an endothelial cell growth medium (ECGM) (EASY ECGM; PELOBiotech, Planegg/Martinsried, Germany) supplemented with 100 U/ml penicillin, 100 μg/ml streptomycin (PAN-Biotech, Aidenbach, Germany), 2.5 μg/ml amphotericin B (PAN-Biotech, Aidenbach, Germany), and 10% FCS (Biochrom, Berlin, Germany), and seeded onto a collagen G (Biochrom, Berlin, Germany)-coated cell culture flask and cultivated at 37°C in an atmosphere of 5% CO_2_ and 95% air under constant humidity. HUVECs were split at a ratio of 1:3 and were used for experimental purposes exclusively as stated in passage 3.

The human monocytic cell line THP-1 and the human T cell line Jurkat were purchased from the German Collection of Microorganisms and Cell Cultures (DSMZ, Braunschweig, Germany). The cells were cultivated at 37°C in an atmosphere of 5% CO_2_ and 95% air under constant humidity in the RPMI 1640 medium (PAN-Biotech) supplemented with 10% FCS (Biochrom, Berlin, Germany), and 100 U/ml penicillin, 100 μg/ml streptomycin (PAN-Biotech, Aidenbach, Germany), and 10% FCS (Biochrom, Berlin, Germany) were used in experiments up to passage 30.

### Cell Adhesion Assay Under Static Conditions

HUVECs at 100% confluency were treated with the indicated concentrations of 6-shogaol or 6-gingerol for 30 min before the cells were activated with LPS (1 μg/ml), IL-1β (5 ng/ml), or TNF (10 ng/ml). After 24 h, THP-1 cells were fluorescence-labeled with CellTracker Green (Thermo Fisher Scientific, Schwerte, Germany), and 100,000 cells per well were allowed to adhere onto an endothelial cell monolayer. The amount of adherent THP-1 or Jurkat cells was measured at 485 nm (ex) and 535 nm (em) using a plate reader (SPECTRAFluor Plus, Tecan, Männedorf, Switzerland).

### Cell Adhesion Assay Under Flow Conditions

HUVECs were seeded into channel slides (µ-Slides I Luer, 0.8 mm; ibidi GmbH, Martinsried, Germany). Subsequently, the HUVECs in channel slides were connected to a pump system (ibidi GmbH, Martinsried, Germany), generating a one-directional flow (5 dyn/cm^2^) on the cells. After 24 h, when the cells had reached 100% confluency, HUVECs were treated with the indicated concentrations of 6-shogaol for 30 min before they were activated by LPS (24 h, 1 μg/ml). Fluorescence-labeled (CellTracker Green) THP-1 cells (8 × 10^5^ cells/ml) were allowed to adhere onto the 6-shogaol-treated and LPS-induced HUVEC monolayer in a one-directional flow of 0.5 dyn/cm^2^. Adherent THP-1 cells on HUVECs were lysed using radioimmunoprecipitation assay (RIPA) buffer, and fluorescence was measured at 485 nm (ex) and 535 nm (em) using a plate reader (SPECTRAFluor Plus, Tecan, Männedorf, Switzerland).

### Transmigration Assay

Potential 6-shogaol-derived effects on leukocyte diapedesis through vascular endothelial cells were determined by performing a transmigration assay. For this, 100,000 HUVECs per well were seeded on collagen-coated Transwell membranes (Corning GmbH HQ, Glendale, AZ, United States, growth area 0.33 cm^2^, 8 µm pore size, polycarbonate) in ECGM. After 24 h, when the cells reached a confluency of 100%, they were treated with the indicated concentrations of 6-shogaol for 30 min before they were activated with LPS (100 ng/ml). After 24 h, 2 × 10^5^ CellTracker Green-labeled THP-1 cells were allowed to transmigrate through the HUVEC monolayer in the direction of an SDF-1 gradient (500 ng/ml; Peprotech, Rocky Hill, NJ, United States) for 2 h. The upper side of the Transwell was exempted from non-transmigrated THP-1 cells using a cotton swab. Transmigrated THP-1 cells on the lower side of the Transwell were lysed in RIPA buffer and quantified by fluorescence measurement at 485 nm (ex) and 535 nm (em) using a plate reader (SPECTRAFluor Plus, Tecan, Männedorf, Switzerland).

### Proliferation Assay

For this assay, 1,500 HUVECs were seeded on collagen-G-coated 96-well plates in ECGM and cultivated for 24 h until they reached a confluency of 30%. Subsequently, the cells were treated with the indicated concentration of 6-shogaol for 72 h. Control cells were fixed using a methanol–ethanol (2:1) solution. After the end of the incubation period, the cells were fixed with methanol–ethanol and stained together with control cells using crystal violet (gentian violet; Sigma-Aldrich, St. Louis, MO, United States). After drying overnight, DNA-bound crystal violet was leached using 20% acetic acid. Absorbance was measured at 590 nm using a plate reader (SPECTRAFluor Plus, Tecan, Männedorf, Switzerland). Obtained data of control cells were subtracted from experimental values before they were further analyzed.

### Directed Migration (Boyden Chamber) Assay

For the analysis of the impact of 6-shogaol on migration in the direction of a serum gradient, a Boyden chamber assay was performed. For this, 100,000 HUVECs were seeded on a collagen-coated Transwell membrane (Corning GmbH HQ, Glendale, AZ, United States; growth area 0.33 cm^2^, 8 µm pore size, polycarbonate) in supplemented ECGM. The cells were allowed to adhere for 4 h to form a confluent layer (100%) before a serum gradient in medium 199 (0%–20%; PAN-Biotech, Aidenbach, Germany) was applied. At the same time, the cells were treated with indicated concentrations of 6-shogaol. HUVECs were allowed to migrate toward the serum gradient for 16 h before they were fixed (methanol:ethanol; 2:1) and stained using a crystal violet (Sigma-Aldrich, St. Louis, MO, United States) solution containing 20% methanol. Non-migrating cells on the upper side of the Transwell insert were thoroughly removed using a cotton swab. After drying overnight, cell-bound crystal violet was leached using 20% acetic acid and quantified by absorption measurement (590 nm) using a plate reader (SPECTRAFluor Plus, Tecan, Männedorf, Switzerland).

### 2D Chemotactic Migration Assay

Further insights into the migratory behavior of cells can be provided by the performance of a 2D chemotactic migration assay. Therefore, 18,000 HUVECs were seeded on chemotaxis slides (ibidi GmbH, Martinsried, Germany) in ECGM and were allowed to adhere for 4 h in order to obtain confluency of about 10% before they were treated with indicated concentrations of 6-shogaol. The cells were allowed to migrate toward a serum gradient (FCS, 20% in medium 199) for 20 h. Live cell imaging in an atmosphere of 5% CO_2_ and 95% air at 37°C, capturing an image every 10 min using a microscope (DM IL LED, Leica Microsystems, Wetzlar, Germany) allowed single-cell tracking. Image quantification of 30 cells per donor and condition using a manual-tracking tool (ImageJ) indicated a detailed 6-shogaol-derived impact on the migration parameters accumulated distance, Euclidean distance, velocity, forward migration index Y (Y:FMI), and directness.

### Spheroid Assay

Endothelial cell spheroids from HUVECs were generated in a supplemented ECGM medium containing 0.24% methylcellulose by using the hanging drop method. Subsequently, the spheroids were embedded into a rat tail collagen I gel. HUVEC sprouting was induced by treating with vascular endothelial growth factor (VEGF; PeproTech, Rocky Hill, NJ, United States) for 20 h in an endothelial cell basal medium (ECBM; PELOBiotech, Planegg/Martinsried, Germany). For the determination of 6-shogaol-derived effects on endothelial cell sprouting, HUVEC spheroids were treated with the indicated concentrations of the compound 30 min prior to VEGF induction. After the incubation period, the spheroids were fixed with 4% Roti Histofix (Carl Roth, Karlsruhe, Germany) before microscopic images were taken (DM IL LED, Leica Microsystems, Wetzlar, Germany). Image analysis using ImageJ (software version 1.49k) allowed the quantification of endothelial cell sprouts.

### Mouse Aortic Ring Assay

Organ (mouse aortae) removal was performed according to the German Animal Welfare Act (§ 4 Tierschutzgesetz) and approval number V54-19c20/21I-FR/Biologicum, Tierhaus Campus Riedberg (Regierungspräsidium Darmstadt, Germany). Animals (C57BL/6 N mice) were housed under a 12 h light/dark cycle with access to food and water *ad libitum*.

To analyze the effect of 6-shogaol on angiogenic processes an *ex vivo* aortic ring assay was performed (Baker et al., 2011). For this, 3–5 week old C57BL/6 mice (male/female) were sacrificed by carbon dioxide (CO_2_) exposure. Subsequently, aortae were dissected, and the adjacent soft tissue was removed before the blood vessels were cut into rings with an average size of 0.5–1 mm. Aortic rings were serum-starved overnight in OPTI-MEM I (Thermo Fisher Scientific, Schwerte, Germany) containing 100 U/ml penicillin and 100 μg/ml streptomycin (PAN-Biotech). The next day aortic rings were embedded into 50 µl rat tail collagen I gel (Corning GmbH HQ; 1.5 mg/ml). After gel solidification, aortic rings were treated with 30 ng/ml murine VEGF (Peprotech, Rocky Hill, NJ, United States) in OPTI-MEM (Thermo Fisher Scientific, Schwerte, Germany), supplemented with 2.5% FCS (Biochrom, Berlin, Germany), 100 U/ml penicillin, and 100 μg/ml streptomycin (PAN-Biotech, Aidenbach, Germany) to induce endothelial sprouting. When initial sprouts were visible, aortic rings were treated with 6-shogaol (30 µM) or vehicle, i.e., 0.03% dimethyl sulfoxide (DMSO), and 30 ng/ml VEGF for additional 3 days. For the analysis of potential 6-shogaol-derived vascular-disruptive effects, endothelial sprouts were allowed to form for 6–9 days, before the aortic rings were treated with 6-shogaol (30 µM) or vehicle (0.03% DMSO) and 30 ng/ml VEGF for 24 or 48 h.

After the respective incubation periods, aortic rings and sprouts were fixed using 4% Roti Histofix (Carl Roth, Karlsruhe, Germany) and permeabilized using Triton-X100 (Carl Roth, Karlsruhe, Germany) two times for 15 min at room temperature. The sprouts were stained using BS-I lectin (FITC, 0.1 mg/ml; lectin from *Bandeiraea simplicifolia* (*Griffonia simplicifolia*); #2895; Sigma-Aldrich; St. Louis, MO, United States) and actin α-smooth muscle (CY3; mouse, anti-mouse, 1:1,000; #C6198; Sigma-Aldrich; St. Louis, MO, United States) at 4°C overnight. Sprouts from murine aortic rings were analyzed using laser confocal scanning microscopy (Zeiss LSM 780; Carl Zeiss, Oberkochen, Germany) and quantified by ImageJ (software version 1.49k; US National Institutes of Health, Bethesda, MD, United States).

### Flow Cytometry

Confluent HUVECs (100% confluency) were treated with indicated concentrations of 6-shogaol. 30 min later, the cells were activated with LPS (1 μg/ml) for 4 h (E-selectin) or 24 h (intercellular adhesion molecule, ICAM-1; vascular cell adhesion molecule, VCAM-1), before they were stained for ICAM-1 (fluorescein isothiocyanate, FITC; mouse, anti-human CD54, 1:33, MCA1615F; Bio-Rad, Hercules, CA, United States), VCAM-1 (phycoerythrin, PE; mouse, anti-human CD106, 1:20, 555647; BD Biosciences, San Jose, CA, United States), or E-selectin (PE, mouse, anti-human CD62E, 1:10, 551145; BD Biosciences, San Jose, CA, United States). Levels of the respective surface protein were determined using flow cytometry (FACSVerse; BD Biosciences, San Jose, CA, United States).

### Western Blot Analysis

Confluent HUVECs (100% confluency) were treated with the indicated concentrations of 6-shogaol for 30 min before they were activated with LPS (1 μg/ml) for 4 (E-selectin) or 24 h (ICAM-1, VCAM-1). Subsequently, the cells were lysed using RIPA buffer containing protease and phosphatase inhibitors. The total protein in each sample was determined using a Pierce BCA Protein Assay Kit (Thermo Fisher Scientific, Schwerte, Germany), and a pyronin-based sample buffer containing sodium dodecyl sulfate (SDS) was added before they were incubated at 95°C for 5 min for denaturation. Twenty-five µg of each sample was subjected to discontinuous SDS-polyacrylamide gel electrophoresis (PAGE) for protein separation, before they were transferred onto a polyvinylidene fluoride (PVDF; Bio-Rad Laboratories, Munich, Germany) membrane by blotting for 1 h at 100 V or 16 h at 30 V (VEGFR2). For blocking of unspecific binding sites, the membranes were incubated for 2 h in 5% Blotto (Carl Roth, Karlsruhe, Germany) or 5% bovine serum albumin (BSA; MilliporeSigma, Burlington, MA, United States) containing 0.1% Tween-20 (Sigma-Aldrich; St. Louis, MO, United States). Subsequently, the membranes were incubated with respective antibodies for detection of the protein of interest: anti-ICAM-1 (rabbit, anti-human, 1:1,000 in 5% Blotto + Tween-20, 4°C overnight, #4915; Cell Signaling/New England Biolabs, Frankfurt am Main, Germany), anti-VCAM-1 (mouse, anti-human, 1:1,000 in 5% Blotto + Tween-20, 4°C overnight, sc-13160; Santa Cruz Biotechnology, Dallas, TX, United States), anti-E-selectin (mouse, anti-human, 1:2,000 in 5% Blotto + Tween-20, 4°C overnight, sc-137054; Santa Cruz Biotechnology), anti-toll-like receptor (TLR) 4 (mouse, anti-human, 1:400 in 5% Blotto + Tween-20, 4°C overnight, sc-293072 Santa Cruz Biotechnology, Dallas, TX, United States), anti-VEGFR2 (rabbit, anti-human, 1:2,000 in 5% BSA + Tween-20, 4°C overnight, #9698; Cell Signaling/New England Biolabs, Frankfurt am Main, Germany), and anti-HO-1 (P249) (rabbit, anti-human, 1:1,000 in 5% BSA + Tween-20, 4°C overnight, #5061; Cell Signaling/New England Biolabs, Frankfurt am Main, Germany). Anti-β-actin-peroxidase (mouse, anti-human, 1:50,000 in 1% BSA + Tween-20, 1 h room temperature, A3854; Sigma-Aldrich, St. Louis, MO, United States) was used to detect β-actin as the loading control. Secondary antibodies were applied to membranes being incubated with non-conjugated primary antibodies: anti-rabbit (goat, 1:2,000 in 5% Blotto + Tween-20, 2 h room temperature, #7074; Cell Signaling/New England Biolabs, Frankfurt am Main, Germany) and anti-mouse, HRP-linked antibody (horse, 1:2,000 in 5% Blotto + Tween-20, 2 h room temperature, #7076; Cell Signaling/New England Biolabs, Frankfurt am Main, Germany). Protein detection was achieved by chemiluminescence measurement, and quantification of specific protein levels in each sample was performed by densitometric analysis using ImageJ (software version 1.49k).

### Immunofluorescence Staining and Microscopy

Confluent HUVECs (100% confluency) on 8-well µ-slides (ibidi GmbH, Martinsried, Germany) were treated with indicated concentrations of 6-shogaol for 24 h before they were activated with LPS (1 μg/ml) for indicated time points. Subsequently, the cells were fixed with 4% Roti Histofix (Carl Roth, Karlsruhe, Germany) before they were permeabilized using 0.2% Triton-X100 (Carl Roth). After blocking unspecific binding sites using 0.2% BSA (MilliporeSigma, Burlington, MA, United States), the cells were incubated in a 0.2% BSA solution containing the primary antibody anti-NFκB-p65 (rabbit, anti-human, 1:400, sc-372; Santa Cruz Biotechnology, Dallas, TX, United States). After 1 h the primary antibody was removed, and the secondary antibody (goat, anti-rabbit, Alexa Fluor 488-conjugated, 1:400, A11008; Thermo Fisher Scientific, Schwerte, Germany) was incubated for 45 min protected from light. Immunofluorescence staining of NFκB-p65 was determined using a Leica DMI6000B fluorescence microscope (Leica Microsystems, Wetzlar, Germany). The use of ImageJ (software version 1.49k; US National Institutes of Health, Bethesda, MD, United States) allowed the quantification of NFκB-p65 translocation.

### Quantitative Polymerase Chain Reaction

Confluent HUVECs (100% confluency) were treated with indicated concentrations of 6-shogaol for 30 min, before they were activated with LPS (1 μg/ml) for 2 h (E-selectin) or 10 h (ICAM-1, VCAM-1). RNA was isolated using the RNeasy mini kit (Qiagen, Hilden, Germany) according to the manufacturer’s instructions, and 1 µg was transcribed into cDNA using the SuperScript reverse transcriptase (Life Technologies, Darmstadt, Germany). The 2^−ΔΔCT^ method was used for quantitative real-time PCR data analysis. GAPDH served as the housekeeping gene, and the following primers were used: E-selectin (forward: 5′-AGA-TGA-GGA-CTG-CGT-GGA-GA-3′; reverse: 5′-GTG-GCC-ACT-GCA-GGA-TGT-AT-3′), VCAM-1 (forward: 5′-CCA-CAG-TAA-GGC-AGG-CTG-TAA-3′; reverse: 5′-GCT-GGA-ACA-GGT-CAT-GGT-CA-3′), ICAM-1 (forward: 5′-CTG-CTC-GGG-GCT-CTG-TTC-3′; reverse: 5′-AAC-AAC-TTG-GGC-TGG-TCA-CA-3′), VEGFR2 (forward: 5′-GTG-ACC-AAC-ATG-GAG-TCG-TGT-3′; reverse: 5′-AGC-TGA-TCA-TGT-AGC-TGG-GAA-3′), and GAPDH (forward: 5′-CCA-CAT-CGC-TCA-GAC-ACC-AT-3′; reverse: 5′-TGA-AGG-GGT-CAT-TGA-TGG-CAA-3′).

### NFκB Promotor Activity Assay

For this assay, 1 × 10^6^ HUVECs (PeloBiotech, Planegg/Martinsried, Germany) per aluminum cuvette were transiently transfected with a *Renilla* luciferase control plasmid pGL4.74[hRluc/TK] and a firefly luciferase reporter plasmid pGL4.32[luc2P/NFκB-RE/Hygro] (Promega, Heidelberg, Germany) at a ratio of 5:10 by electroporation (Amaxa nucleofector 2b device; Lonza, Basel, Switzerland). Twenty-four h after transfection, when HUVECs reached a density of 100% (confluency), the cells were treated with the indicated concentrations of 6-shogaol or 6-gingerol for 6 h before they were activated by LPS for 4 h (NFκB). Afterward, HUVECs were exposed to passive lysis according to the manufacturer’s instructions, and NFκB promotor activity was determined using the Dual-Luciferase Reporter Assay System (Promega, Heidelberg, Germany) using a luminometer (SPECTRAFluor Plus, Tecan, Männedorf, Switzerland).

### Statistical Analysis

All experiments were performed with at least three different HUVEC or aortae donors (n). The actual number of n is stated in the respective figure legend. Statistical analyses were performed using GraphPad Prism version 5.0 (San Diego, CA, United States) by applying one-way ANOVA and Tukey’s *post hoc* test. For the *ex vivo* assay, an unpaired Student’s t-test followed by Welch’s correction was used. Data are expressed as mean ± standard deviation (SD) and considered as statistically significant when *p* ≤ 0.05.

## Results

### 6-Shogaol Strongly and Significantly Reduces Leukocyte–Endothelial Cell Interactions Upon Lipopolysaccharide-Induced Endothelial Cell Activation

Studying the potential of 6-shogaol to interfere with this process in endothelial cells (ECs), we compared the impact of the compound on the adhesion of the monocytic THP-1 cell line onto compound-treated and TNF-, IL-1β-, or LPS-activated HUVECs under static conditions.

In our study, only HUVECs were treated with 6-shogaol or 6-gingerol (Supplementary Figure S1). Analyzing potential cytotoxic effects, we found that up to 30 µM of 6-shogaol incubated over a period of 24, 48, and 72 h did not impair endothelial cell viability nor membrane integrity (Supplementary Figures S2A,B). Therefore, for experimental purposes, concentrations of no more than 30 µM were used. For the exclusion of potential cytotoxic effects of 6-gingerol, HUVECs were treated with increasing concentrations for 24 and 48 h. Up to 100 µM did not impair the cell viability (Supplementary Figure S2C).

**FIGURE 1:**
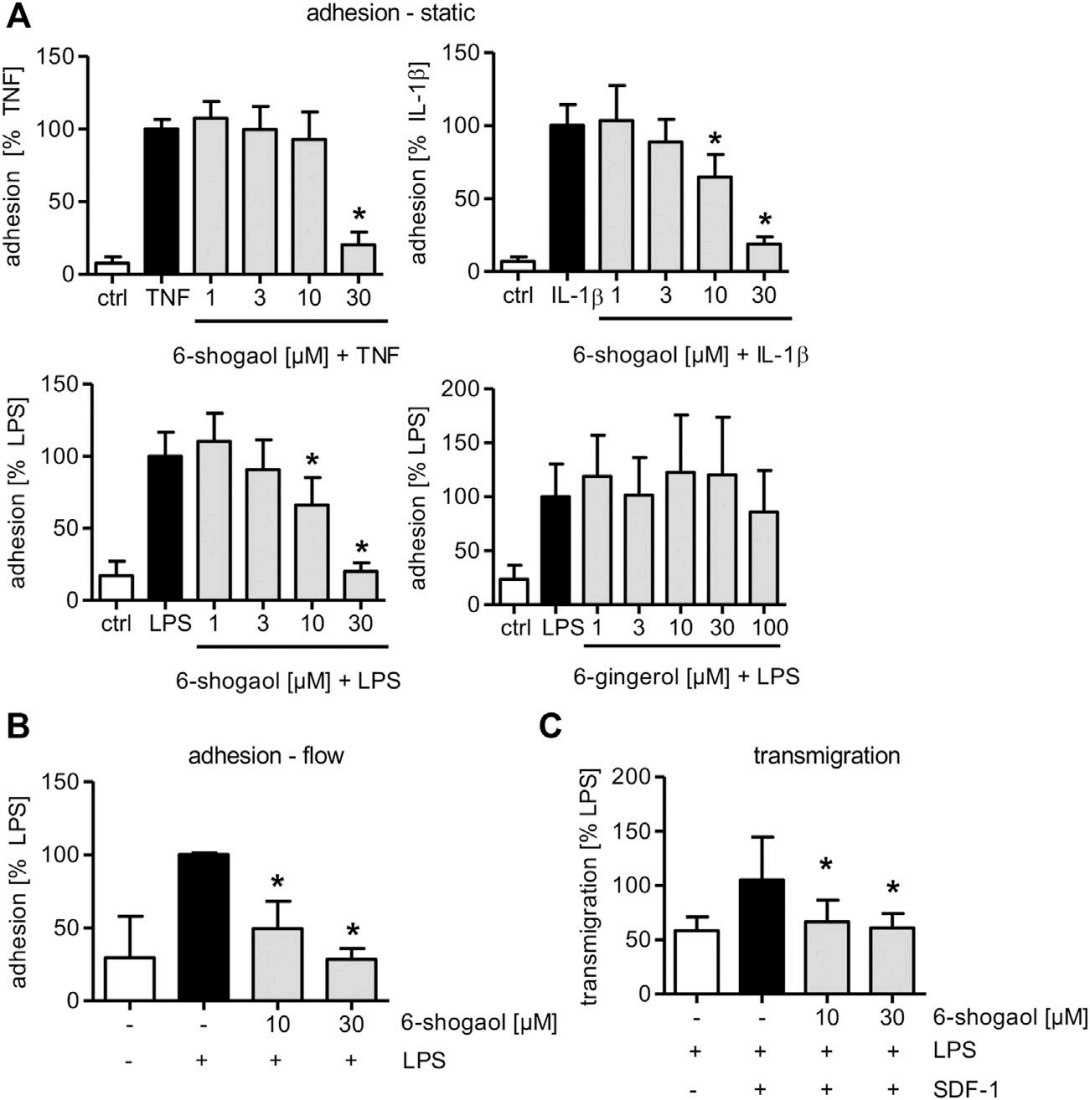
6-Shogaol significantly attenuates leukocyte adhesion and transmigration onto/through an LPS-induced HUVEC monolayer, while 6-gingerol does not impair leukocyte–endothelial cell interactions. Confluent HUVECs were treated with indicated concentrations of **(A–C)** 6-shogaol or **(A)** 6-gingerol for 30 min before they were activated for 24 h by **(A)** TNF (10 ng/ml), **(A)** IL-1β (5 ng/ml), or **(A–C)** LPS (1 μg/ml). Subsequently, fluorescence-labeled THP-1 cells were allowed to adhere under **(A)** static or **(B)** flow conditions. Adhesion of THP-1 cells was measured using a plate reader. **(C)** To determine 6-shogaol-derived effects on transmigration of THP-1 cells through an endothelial cell monolayer, confluent HUVECs on Transwell inserts were treated with the indicated concentrations of 6-shogaol for 30 min before they were activated with LPS (1 μg/ml) for 24 h. CellTracker Green-labeled THP-1 cells were allowed to transmigrate through a HUVEC monolayer toward an SDF-1 gradient. Transmigrated THP-1 cells were quantified by fluorescence measurement using a plate reader. Data are expressed as mean ± SD; **(A)** *n* = 4/4/4/5; *p ≤ 0.05 vs. TNF/IL-1β/LPS; **(B)** *n* = 3; *p ≤ 0.05 vs LPS; **(C)** *n* = 3; *p ≤ 0.05 vs. LPS/SDF-1.

6-Shogaol at a concentration of 30 µM significantly reduced the adhesion of THP-1 cells onto a TNF-, IL-1β-, or LPS-triggered HUVEC monolayer under static conditions (Figure 1A). When TNF was used as a pro-inflammatory stimulus, a concentration of 30 µM of 6-shogaol was able to almost fully block the adhesion. In HUVECs that were treated by IL-1β or LPS, 6-shogaol was already active at a concentration of 10 µM. To determine if 6-shogaol was able to reduce the adhesion of leukocytes onto an LPS-induced HUVEC monolayer under the flow conditions, a pump system was used to mimic the blood flow. 6-shogaol was able to reduce the adhesion of THP-1 cells onto LPS-activated HUVECs significantly at 10 and 30 µM. Of note, treatment of HUVECs with 30 µM 6-shogaol completely blocked the adhesion of THP-1 cells under flow conditions (Figure 1B). In addition to 6-shogaol, 6-gingerol has been described as one of the main bioactive ingredients of ginger rhizomes and represents the hydrated precursor of 6-shogaol missing the Michael acceptor moiety. Interestingly, the application of up to 100 µM of 6-gingerol did not reduce THP-1 adhesion onto LPS-induced HUVECs (Figure 1A).

Furthermore, the adhesion of the leukemia cell line Jurkat with T-lymphoblast-like phenotype was significantly reduced in LPS-activated HUVECs when treated with 30 µM 6-shogaol (Supplementary Figure S3). Moreover, and of high importance, 6-shogaol reduced the transmigration of THP-1 cells through LPS-triggered HUVECs in the direction of an SDF-1-gradient. This effect was already significant at 10 µM and completely blocked by 30 µM 6-shogaol (Figure 1C).

### 6-Shogaol Attenuates the Levels of Cell Adhesion Molecules, and the Effect Is Most Prominent for Lipopolysaccharide-Activated Endothelial Cells

**FIGURE 2:**
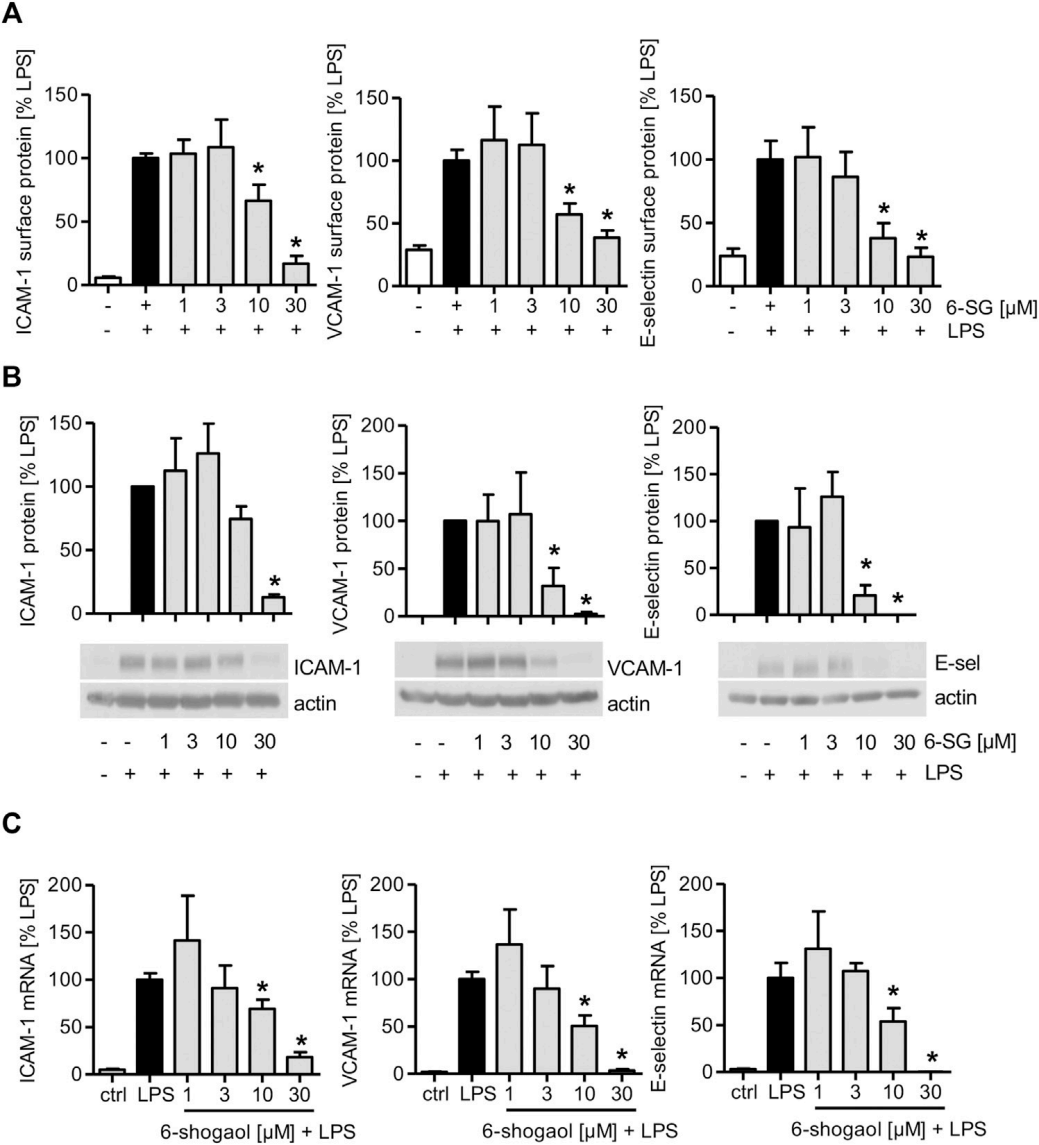
LPS-induced CAM levels in HUVECs are downregulated by 6-shogaol in a concentration-dependent manner. Confluent HUVECs were treated with indicated concentrations of 6-shogaol. After 30 min, the cells were activated by LPS (1 μg/ml) for **(A,B)** 4 h (E-selectin) or 24 h (ICAM-1 and VCAM-1) to determine protein levels and for **(C)** 2 h (E-selectin) or 12 h (ICAM-1 and VCAM-1) for mRNA expression. **(A)** Surface protein levels of ICAM-1, VCAM-1, and E-selectin were determined by flow cytometry, and **(B)** total protein levels of CAMs were analyzed by Western blot analysis. Actin served as the loading control. One representative blot is shown. **(C)** mRNA data were obtained by qPCR. GAPDH served as the housekeeping gene. 6-SG: 6-shogaol. Data are expressed as mean ± SD; **(A,C)** *n* = 3; **(B)** *n* = 4/4/3; *p ≤ 0.05 vs. LPS.

As 6-shogaol reduced the interaction of THP-1 and Jurkat cells and activated HUVECs, the levels of endothelial cell adhesion molecules (CAMs) on the endothelial surface, their total amount, and mRNA expression were determined. 6-Shogaol significantly reduced TNF-induced ICAM-1, VCAM-1, and E-selectin levels on the endothelial cell surface (Supplementary Figure S4A). The determination of 6-shogaol-derived effects revealed that also the total protein levels of CAMs were reduced, and the most prominent effect was observed at 30 µM (Supplementary Figure S4B). The analysis of mRNA levels revealed that increasing concentrations of 6-shogaol reduced the gene expression of ICAM-1, VCAM-1, and E-selectin in a concentration-dependent manner (Supplementary Figure S4C). Similar effects were observed after 6-shogaol treatment, when HUVECs were activated with the inflammatory cytokine IL-1β. Here, the most prominent inhibitory effects were also detectable at 30 µM (Supplementary Figures S5A–C). Interestingly, when LPS was used for the inflammatory activation of HUVECs, 6-shogaol significantly reduced surface levels of ICAM-1, VCAM-1, and E-selectin already at 10 µM. Of note, the application of 30 µM was able to completely block the surface levels of CAMs (Figure 2A). In addition, the effects on total protein and mRNA levels were much stronger for the activation with LPS compared to the application of the cytokines TNF or IL-1β. Here, 6-shogaol significantly reduced CAM levels at 10 µM and blocked their expression at 30 µM (Figures 2B,C).

### 6-Shogaol Blocks the NFκB Promotor Activity, While 6-Gingerol Does Not Affect the Transcription Factor

**FIGURE 3:**
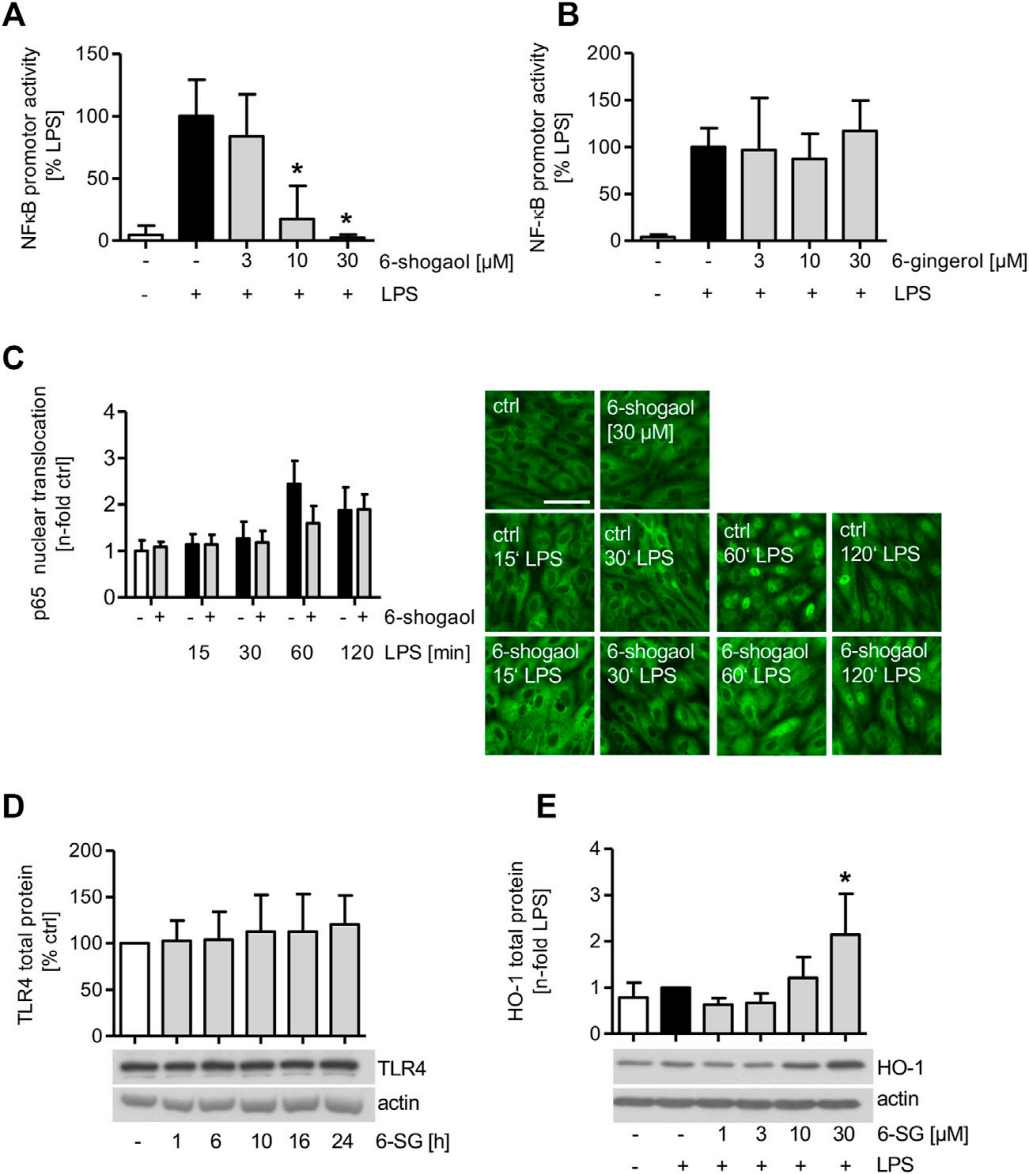
LPS-induced NFκB promotor activity is blocked by 6-shogaol but remains unimpaired by 6-gingerol. **(A,B)** 1 × 10^6^ HUVECs per aluminum cuvette were transiently transfected with a *Renilla* luciferase control plasmid pGL4.74[hRluc/TK] and firefly luciferase reporter plasmid pGL4.32[luc2P/NF-κB-RE/Hygro] using a Nucleofector 2b device. 24 h after transfection, the cells were treated with indicated concentrations of **(A)** 6-shogaol or **(B)** 6-gingerol for 6 h before NFκB promotor activity was induced by LPS (1 μg/ml) for an additional 4 h. NFκB promotor activity was measured using a luminometer. **(C)** Confluent HUVECs were treated with 30 µM of 6-shogaol before the cells were activated with LPS (1 μg/ml) for the indicated time points. Subsequently, the cells were stained for p65, and fluorescence microscopy images were analyzed using ImageJ software. For each condition, one representative image out of four independently performed experiments is shown. Scale bar represents 50 µm. **(D)** Confluent HUVECs were treated with 30 µM of 6-shogaol for indicated time points. Protein levels of TLR4 were determined by Western blot analysis. Actin served as the loading control. One representative blot of four independently performed experiments is shown. **(E)** Confluent HUVECs were treated with indicated increasing concentrations of 6-shogaol and were induced with LPS (1 μg/ml) for 24 h before the samples were subjected to Western blot analysis for HO-1 total protein. Actin served as the loading control. One representative Western blot is shown. 6-SG: 6-shogaol. Data are expressed as mean ± SD; **(A,B)** *n* = 3; *p ≤ 0.05 vs. LPS; **(C,D)** *n* = 4; **(E)** *n* = 5; *p ≤ 0.05 vs. LPS.

The effect of 6-shogaol on the activity of NFκB was analyzed in LPS-induced HUVECs. Moreover, to compare the effect of 6-gingerol on the NFκB-signaling cascade, HUVECs were treated with the indicated concentrations of 6-gingerol before they were analyzed for the LPS-induced promotor activity of the transcription factor. Interestingly, the promotor activity of NFκB was strongly downregulated by 6-shogaol in a concentration-dependent manner and completely blocked by a compound concentration of 30 μM, while 6-gingerol did not impair the promotor activity of the transcription factor (Figures 3A,B). Moreover, LPS-induced inflammatory target genes of NFκB were downregulated by 6-shogaol. The mRNA levels of the pro-inflammatory cytokines IL-6 and IL-8 were significantly reduced in a concentration-dependent manner (Supplementary Figure S6A). The mRNA expression of the LPS-triggered chemokine C-X-C motif chemokine ligand 12 (CXCL12) was significantly reduced at 30 µM 6-shogaol (Supplementary Figure S6B). Interestingly, mRNA levels of the anti-inflammatory cytokine IL-10 remained unimpaired (Supplementary Figure S6C).

Mitogen-activated protein kinases (MAPKs) were implicated in the NFκB-signaling cascade, and Western blot analysis revealed that the LPS-induced c-Jun N-terminal kinase (JNK) phosphorylation was markedly reduced upon 6-shogaol (30 µM) treatment, while the LPS-triggered p38 activation was not impaired (Supplementary Figure S6D).

### HO-1 Protein Levels are Increased Upon 6-Shogaol Treatment, While Nuclear Translocation of the NFκB Subunit p65 is Only Marginally Reduced

As the 6-shogaol-derived inhibition of LPS-induced NFκB promotor activity might be attributed to an impairment of nuclear translocation, the amount of the NFκB subunit p65 was analyzed in HUVEC nuclei after the treatment with 6-shogaol (30 µM) by immunofluorescence staining (Figure 3C). Interestingly, only after 1 h of LPS induction, 6-shogaol reduced the nuclear translocation of p65 by 35%. Analyzing the effect of 6-shogaol (30 µM) on the toll-like receptor 4 upstream the LPS-activated NFκB-signaling cascade, we found that the protein levels remained unimpaired (Figure 3D).

HO-1 protein levels were markedly increased in LPS-stimulated HUVECs upon 6-shogaol (30 µM) treatment (Figure 3E).

### 6-Shogaol Attenuates Angiogenesis-Related Endothelial Cell Functions *In Vitro*

**FIGURE 4:**
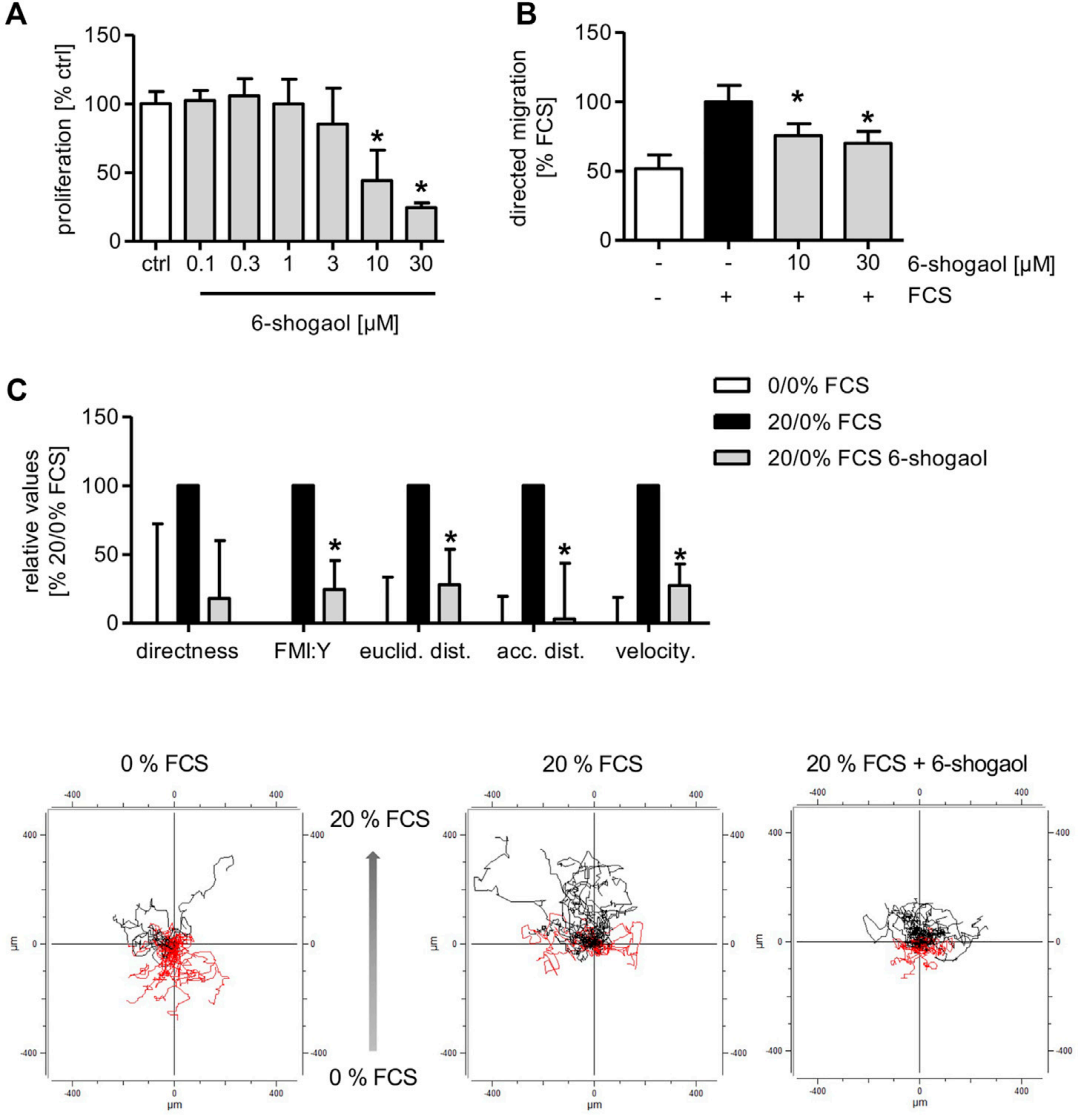
6-Shogaol reduces endothelial cell proliferation and migration. **(A)** 1,500 HUVECs per well were seeded on collagen-G-coated 96-well plates. 24 h later, the cells were treated with 6-shogaol at the indicated concentrations for 72 h. Subsequently, the cells were stained using crystal violet. DNA-bound crystal violet was leached using acetic acid (20%), and absorbance of the dye was measured in a plate reader at 590 nm. **(B)** 100,000 HUVECs were seeded onto Transwell inserts. Subsequently, the cells were treated with indicated concentrations of 6-shogaol, and a serum gradient was generated by the addition of 20% FCS to the lower chamber. The cells were allowed to migrate toward the FCS gradient for 16 h. **(C)** 18,000 HUVECs were seeded on chemotaxis slides, treated with 6-shogaol (30 µM), and allowed to migrate in the direction of a serum (20%) gradient for 20 h. Using phase-contrast microscopy, images were taken every 10 min, and 30 cells per condition were analyzed using ImageJ. Data are expressed as mean ± SD; **(A–C)** *n* = 3; **(A)** *p ≤ 0.05 vs. ctrl; **(B,C)** *p ≤ 0.05 vs. FCS ctrl.

The application of 6-shogaol resulted in a concentration-dependent reduction of HUVEC proliferation with an IC_50_ of 8 µM (Figure 4A). Moreover, the directed HUVEC migration toward a serum gradient was significantly reduced upon 6-shogaol treatment at 10 and 30 µM (Figure 4B). Next, we aimed at gaining detailed information about the impact of the ginger constituent on endothelial cell migration in a 2D chemotaxis assay *via* live-cell imaging. Here, HUVECs were allowed to migrate in the direction of a serum gradient over a period of 20 h. By single-cell tracking, detailed data were obtained about the cell motility upon 6-shogaol (30 µM) treatment. Indeed, the main characteristics of endothelial cell migration were impaired as indicated by significantly reduced directness and velocity. Moreover, 6-shogaol attenuated Euclidean and accumulated distances. The most prominent effect was exposed on the forward migration index (FMI:Y) (Figure 4C).

**FIGURE 5:**
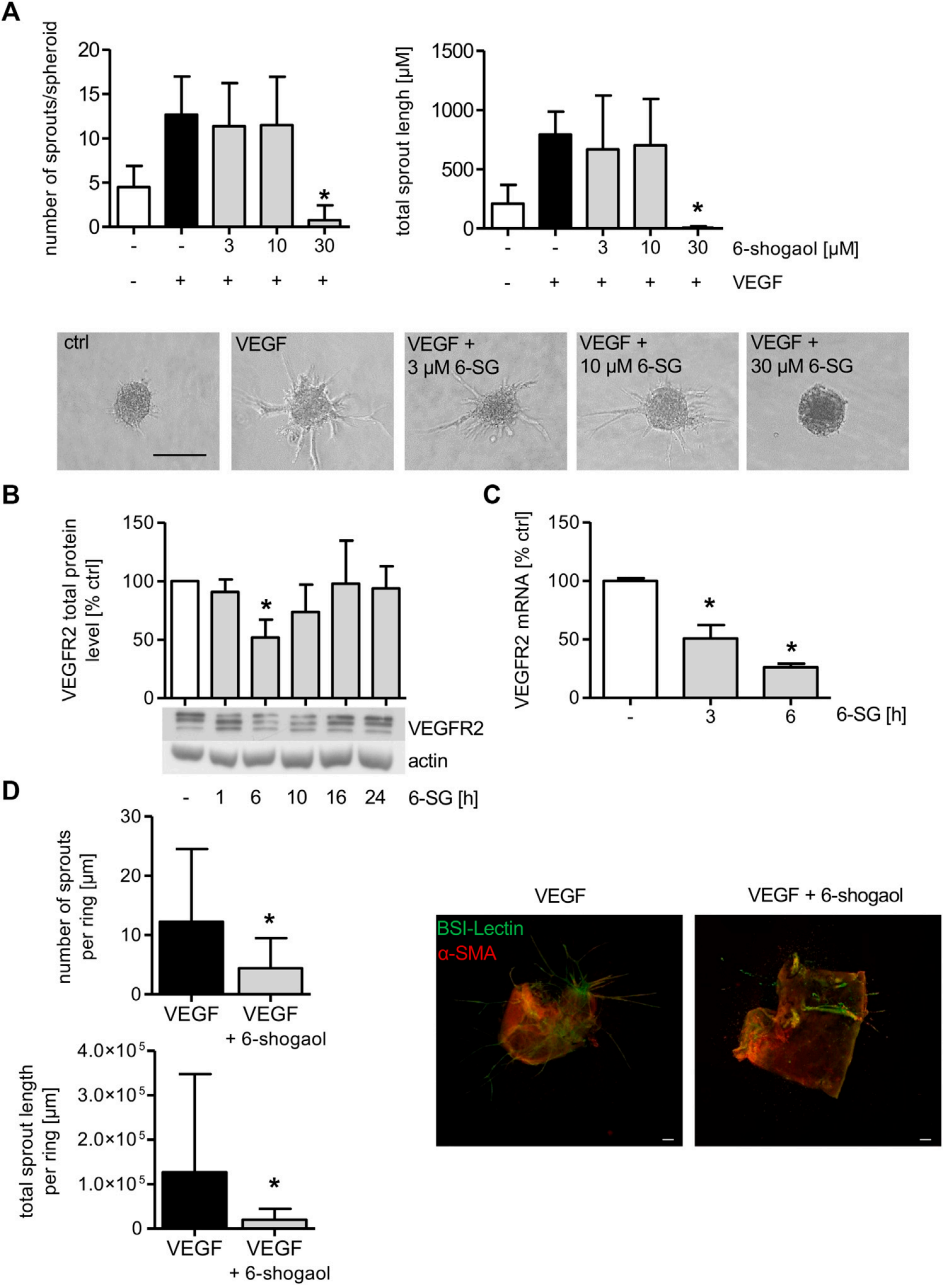
Formation of endothelial sprouts from HUVEC spheroids or mouse aortic rings is significantly attenuated by 6-shogaol. **(A)** HUVEC spheroids comprised 400 cells that were embedded into a rat tail collagen I gel. The spheroids were treated with indicated concentrations of 6-shogaol for 30 min before endothelial sprouting was induced by VEGF (10 ng/ml). 20 h later, the spheroids were fixed with 4% formaldehyde, and microscopic images were taken. Image quantification was performed using ImageJ. Scale bar represents 100 µm. One representative image for each condition is shown. **(B,C)** Confluent HUVECs were treated with 6-shogaol (30 µM) for indicated time points. **(B)** Total protein levels of VEGFR2 were determined by Western blot analysis. Actin served as the loading control. One representative blot is shown. **(C)** VEGFR2 mRNA data were obtained by qPCR. GAPDH served as the housekeeping gene. **(D)** Mouse aortic rings were embedded into a rat tail collagen I gel and stimulated with VEGF (30 ng/ml). After the formation of initial sprouts, the aortic rings were treated with 6-shogaol (30 µM) or vehicle (0.03% DMSO) for 3 days. Aortic rings were fixed and stained with BS-I lectin (green) and for α-smooth muscle actin (red). Laser confocal scanning microscopy and ImageJ allowed the quantification of sprout number and length. Scale bar represents 100 µm, and one representative image is shown. 6-SG: 6-shogaol. Data are expressed as mean ± SD; **(A)** *n* = 3; *p ≤ 0.05 vs. VEGF; **(B)** *n* = 3; **(C)** *n* = 3; *p ≤ 0.05 vs. ctrl; **(D)** *n* = 3; *p* ≤ 0.05 vs. VEGF.

To determine the effect of 6-shogaol on endothelial cell sprouting, spheroids were formed from HUVECs, and the cells were allowed to sprout in a 3D rat tail collagen I gel after VEGF induction. Cells in spheroids were treated with the indicated concentrations of 6-shogaol for 30 min, before VEGF was applied to trigger sprout formation. As depicted in Figure 5A, VEGF successfully induced sprout formation as demonstrated by a strong increase in sprout number and sprout length. The administration of 3 and 10 µM 6-shogaol only slightly reduced the formation of new sprouts. However, 30 µM of 6-shogaol completely blocked endothelial cell sprouting as indicated by a strongly inhibited number of sprouts and reduced sprout length. Of note, the number and length of VEGF-activated sprouts were much lower in 6-shogaol (30 µM)-treated HUVEC spheroids than in the untreated control implicating a compound-induced loss of endothelial cell functions necessary for sprouting processes.

As the VEGF-induced endothelial cell sprouting from HUVEC spheroids was completely blocked by 6-shogaol (30 µM), the effect of the ginger constituent on the total VEGFR2 protein was analyzed. 6-Shogaol (30 µM) markedly reduced the levels of the receptor after 6 and 10 h of treatment, and this impact ceased after 16 h of incubation (Figure 5B). qPCR analysis revealed that the mRNA expression of VEGFR2 was significantly reduced after 3 and 6 h. This indicates that the 6-shogaol-derived downregulation of VEGFR2 might, at least in part, be ascribed to attenuated mRNA levels (Figure 5C).

### 6-Shogaol Exerts Antiangiogenic Properties and Disrupts Pre-Existing Vessels *Ex Vivo*

To obtain detailed information about the 6-shogaol-derived antiangiogenic potential in a physiologically more relevant model, a mouse aortic ring assay was performed. As depicted in Figure 5D, angiogenic sprouts were clearly formed in the vehicle-treated group. FITC-BS-I lectin (green) staining visualizes endothelial sprouting and CY3 (red) indicates actin α-smooth muscle-positive cells. The administration of 6-shogaol resulted in a strong and significant inhibition of VEGF-induced angiogenic sprout formation as indicated by a significantly attenuated number of sprouts and total sprout length per aortic ring.

**FIGURE 6:**
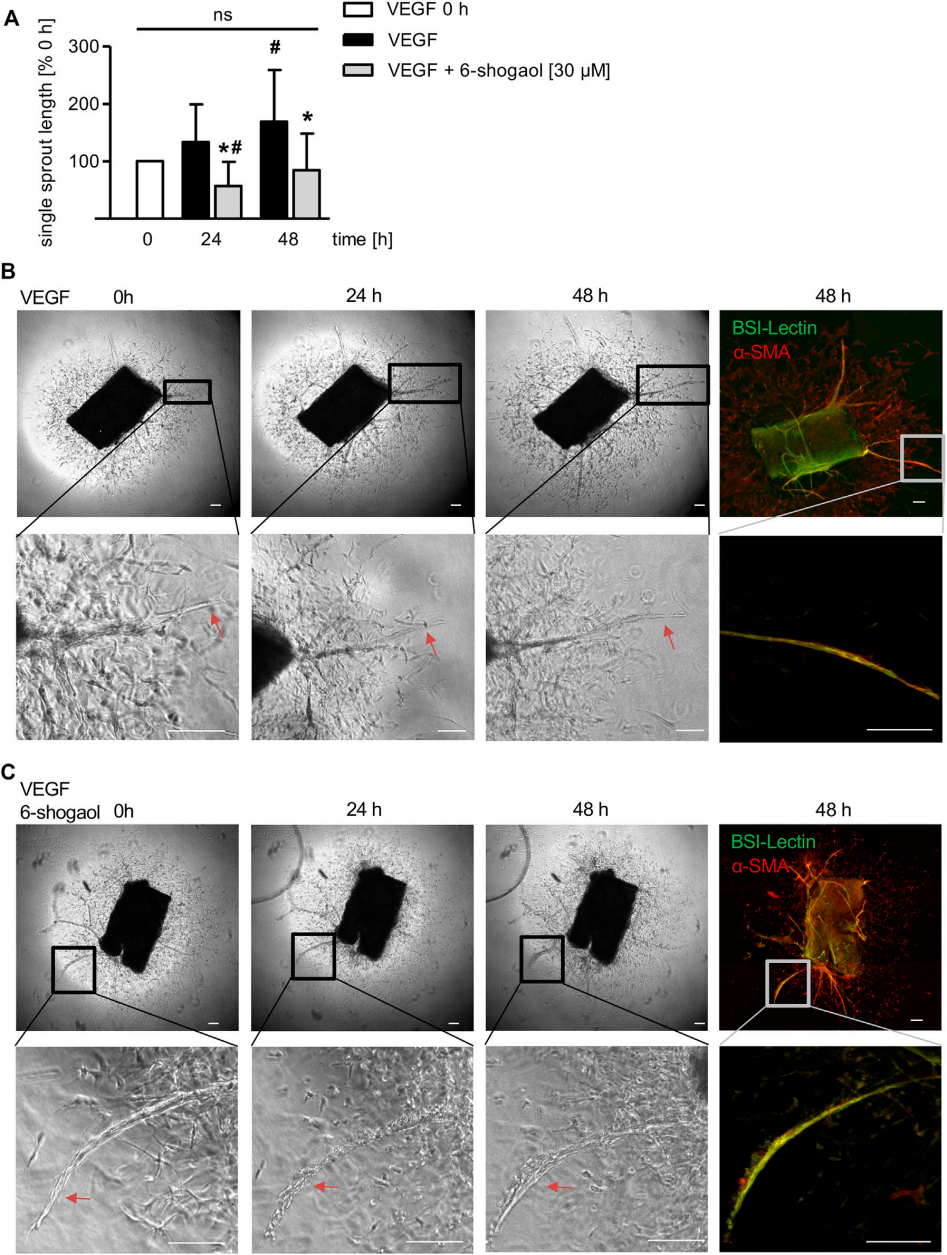
6-Shogaol exhibits a vascular-disruptive impact on angiogenic sprouts from murine aortae. Murine aortic rings were embedded into a rat tail collagen I gel. The rings were allowed to form VEGF-induced (30 ng/ml) angiogenic sprouts for 6–9 days before they were treated with 6-shogaol for 24 and 48 h. Aortic rings were fixed and stained for BS-I lectin (green) and α-smooth muscle actin (red). **(B,C)** Laser confocal scanning microscopy, phase contrast microscopy, and ImageJ allowed **(A)** the quantification of sprout length. Higher magnification shows the effect of **(C)** 6-shogaol or **(B)** vehicle (0.03% DMSO) on already existing vessels. Scale bar represents 100 μm, one representative image is shown, and red arrows highlight the compound-derived impact on preexisting vessels. ns, not significant. Data are expressed as mean ± SD; **(A)** *n* = 3; *p ≤ 0.05 vs. VEGF; ^#^ *p* ≤ 0.05 vs. VEGF 0 h.

Interestingly, 6-shogaol was also able to interfere with already existing neo-vessels formed from VEGF-induced murine aortic rings (Figure 6). The sprout formation in the VEGF control was significantly increasing after 48 h compared to 0 h (Figures 6A,B). Already after 24 h of 6-shogaol treatment (30 µM), the compound disrupted existing sprouts, and this effect was significant compared to the control group treated with VEGF alone and, moreover, to the starting point of administration (0 h) as indicated by the reduction of single sprout length (Figure 6A). Of note, after 48 h angiogenic sprouts were restituted (Figures 6A,C).

## Discussion

In traditional medicine, the use of ginger rhizomes to treat diseases is versatile, and numerous preclinical studies identified ginger to exhibit anticancer, antioxidative, antinausea, anti-inflammatory, and cardioprotective actions (Ali et al., 2008; Nicoll and Henein, 2009; Palatty et al., 2013; Al-Nahain et al., 2014; Prasad and Tyagi, 2015). In the last century, the main pungent principles 6-gingerol and its dehydrated form 6-shogaol—the latter one containing a Michael acceptor moiety—were identified as bioactive and were extensively investigated. In respect of anti-inflammatory actions, several approaches were conducted to compare the effects of both compounds, asserting a greater impact of 6-shogaol over 6-gingerol within this context (Bischoff-Kont and Fürst, 2021).

The vascular endothelium plays a crucial role in the process of acute and chronic inflammation as it represents a barrier for immune cells. Moreover, in the course of inflammation, angiogenic processes are promoted to form new vessels leading to the acceleration and extension of the inflammatory response. Although the effect of 6-shogaol as anti-inflammatory substance has been identified in *in vivo* models and *in vitro* in leukocytes and other cell types, studies on the vascular endothelium are scarce. Therefore, we aimed to analyze the effects of 6-shogaol on crucial steps of inflammation- and angiogenesis-related endothelial cell functions. 6-Shogaol strongly and significantly reduced the adhesion of leukocytes onto LPS- or cytokine-induced HUVECs by the downregulation of endothelial CAMs *in vitro*. Interestingly, 6-shogaol exhibited the most prominent impact on LPS-activated HUVECs by completely blocking THP-1 cell adhesion to control levels in a static system and under flow conditions. A study by Wang et al. (2013) demonstrated strong inhibitory effects of 6-shogaol in oxidized LDL-induced HUVECs. Due to the strong impact on LPS-evoked leukocyte adhesion, we focused on further investigations on the actions of 6-shogaol on LPS-activated ECs. 6-Shogaol strongly and significantly attenuated the transmigration of THP-1 cells through an LPS-activated HUVEC monolayer. In a study by Levy et al. (2006), the administration of 6-shogaol significantly reduced the migration of leukocytes into the joints of rats in a complete Freund’s adjuvant-induced arthritis model. An *in vivo* study employing a murine acute kidney injury model observed a significant reduction of kidney injury and neutrophil infiltration into the kidneys after the administration of 6-shogaol and increased mRNA and protein levels of HO-1 (Han et al., 2019). Interestingly, the application of an HO-1 inhibitor completely reversed this effect, indicating the involvement of this enzyme in the anti-inflammatory actions of 6-shogaol. Additionally, the same group demonstrated an increase of this protein in the human kidney cell line HK2 as well as in mouse kidney tissue samples (Han et al., 2019). Similar to these findings, in our study, the protein levels of HO-1 were significantly increased by 30 µM 6-shogaol and LPS in HUVECs. Moreover, a study by Seldon et al. (2007) examined the regulation of cell adhesion molecule levels in endothelial cells isolated from *HMOX* ^−/−^ mice vs. *HMOX* ^+/+^ mice. Basal and TNF-induced expression of VCAM-1, ICAM-1, and E-selectin were increased in *HMOX* ^−/−^ mice vs. *HMOX* ^+/+^ mice, which indicates that the blocking of HO-1 might result in increased levels of CAMs. This effect might, at least in part, counteract the inhibiting effect of 6-shogaol. A study by Liu et al. (2015) shows that 6-shogaol effectively inhibited the proliferation of rat aortic vascular smooth muscle cells with an IC_50_ of 2.7 µM without inducing cytotoxicity. Interestingly, 6-shogaol lost its antiproliferative feature when an HO-1 inhibitor was applied. These data give hints that the inhibition of HO-1 might reverse the effects of 6-shogaol on inflammation- and angiogenesis-related processes.

Concomitant to the upregulation of cytoprotective enzyme HO-1, the LPS-induced promotor activity of NFκB was completely blocked in HUVECs after the treatment with 6-shogaol. This exuberant effect resulted in the downregulation of endothelial CAM mRNA expression and the subsequent attenuation of total and surface protein levels of ICAM-1, VCAM-1, and E-selectin, which constitute prominent mediators of leukocyte–EC interactions and, therefore, might be, at least in part, responsible for the reduced leukocyte adhesion onto and transmigration through vascular endothelial cells. Several *in vivo* and *in vitro* studies reported a 6-shogaol-derived inhibitory impact on the NFκB-signaling cascade, including the reduction of nuclear translocation of p50 and p65 subunits (Pan et al., 2008; Han et al., 2017; Annamalai and Suresh, 2018; Han et al., 2019). Interestingly, the nuclear translocation of the NFκB subunit p65 was only reduced by 35% after 6-shogaol treatment and 1 h of LPS activation, indicating a different mechanism of 6-shogaol for this distinct impact on the NFκB promotor activity. Nevertheless, our findings suggest that the downregulation of the promotor activity of the transcription factor might be, at least in part, responsible for the reduction of endothelial CAM and IL-6 and IL-8 expressions. Similar data were obtained in animal and *in vitro* studies demonstrating the reduction of soluble VCAM-1 protein as well as the attenuation of further NFκB target genes, including ICAM-1 in proximal tubule cells and proinflammatory cytokines in HaCaT or human mast cells (Levy et al., 2006; Guahk et al., 2010; Sohn et al., 2013; Han et al., 2019). Based on the Michael system of 6-shogaol, a potential impact on the p65 subunit of NFκB by targeting cysteine residues, for example, by alkylation, can be suggested. This might be followed by the inhibition of the binding of p65 to regulatory regions of the DNA. A study by Garcia-Pineres et al. (2001) employed a mutant lacking different cysteines in p65. The application of the anti-inflammatory p65-alkylating sesquiterpene lactone parthenolide (NFκB inhibitor containing a Michael acceptor) had no effect on the DNA-binding capacity of the p65 subunit in p65 mutants that lack a certain cysteine residue. Therefore, it could indeed be hypothesized that 6-shogaol might suppress the DNA-binding capacity of p65, and this, at least in part, might be responsible for the downregulation of CAM gene expression. Further experiments are necessary to test this hypothesis. Helenalin, a sesquiterpene lactone found in *Arnica montana* L., exhibits anti-inflammatory properties through the inhibition of the NFκB-signaling cascade by directly targeting p65 (Lyss et al., 1997). Based on the Michael system of this compound, studies have been conducted demonstrating that helenalin alkylates p65, which in turn leads to the inhibition of NFκB-p65 signaling. The translocation of p65 was not significantly impaired by helenalin. Likewise, in our study, only a slight impact of p65 nuclear translocation was detected after 6-shogaol treatment and 1 h of LPS activation. Since 6-shogaol has been shown to alkylate cysteine residues of Kelch-like ECH-associated protein 1 (Keap1) and since the NFκB p50 and p65 subunits contain cysteine residues in their DNA binding domains, we suggest that 6-shogaol might inhibit the NFκB-signaling cascade in the nucleus of endothelial cells by Michael acceptor-mediated alkylation.

While the literature reports contradictory results for the impact of 6-shogaol on MAPK activation, we found that, similar to the findings of Sohn et al. (2013), the ginger constituent reduced the LPS-triggered phosphorylation of JNK in endothelial cells, while the activation of p38 remained unimpaired. Interestingly, upstream of the NFκB-signaling cascade, the total protein levels of the LPS receptor TLR4 were not affected by 6-shogaol. Nevertheless, there is evidence that 6-shogaol might interfere with the dimerization process of TLR4 and, therefore, impair the down-stream signaling after LPS treatment (Ahn et al., 2009). Of note, 6-gingerol, lacking the Michael acceptor moiety, did neither impair the LPS-induced promotor activity of NFκB nor interfere with THP-1 cell adhesion onto LPS-induced and compound-treated ECs. In accordance with our findings, studies comparing the impact of both bioactive ginger constituents, 6-shogaol and 6-gingerol, within the scope of inflammation-related cell functions in other cell types found that 6-shogaol exhibited a much stronger anti-inflammatory activity than 6-gingerol (Dugasani et al., 2010; Guahk et al., 2010; Ho and Chang, 2018).

Moreover, our study demonstrates the successful 6-shogaol-induced impairment of angiogenesis-related cell functions as indicated by the reduction of endothelial cell proliferation, chemotactic migration, and *in vitro* and *ex vivo* sprouting. Liu et al. (2015) reported the inhibition of vascular smooth muscle cell proliferation with an IC_50_ of 1 µM. In accordance with this, 6-shogaol was able to reduce the proliferation of HUVECs with an IC_50_ of 8 µM without exerting cytotoxic effects up to 30 µM over an incubation period of 72 h. In our study, we present the impact of 6-shogaol on chemotactic migration, which represents one of the key features of angiogenesis. Here, 6-shogaol significantly downregulated the migration of ECs in a Boyden chamber toward a serum gradient. Moreover, we tried to gain deeper insights into the anti-migratory impact of 6-shogaol by the performance of a chemotaxis assay. 6-shogaol significantly reduced chemotactic parameters, such as forward migration, Euclidean and accumulated distance as well as velocity. To the best of our knowledge, this is the first report describing this impact of 6-shogaol on this crucial feature of angiogenesis. A study by Weng et al. (2012) employed a tube formation assay using Matrigel and demonstrated that 6-shogaol reduced the formation of tube-like structures. In concert with this, 30 µM of 6-shogaol completely blocked the formation of VEGF-induced capillary-like sprouts from HUVEC spheroids embedded into a 3D collagen gel. Interestingly, analyzing the impact of 6-shogaol on VEGFR2, the main mediator that controls angiogenic processes in ECs, we found that the protein levels and mRNA expression were significantly downregulated. This effect might be specific as the protein levels of TLR4 remained unimpaired by 6-shogaol. To mimic the multi-cellular *in vivo* situation, a mouse aortic ring assay was performed. BS-I lectin staining indicated the existence of ECs and their angiogenic differentiation. The additional staining of smooth-muscle actin-positive cells demonstrates the successful intercellular interaction of cell types being necessary for the development of maturated angiogenic structures. In concert with the findings of the HUVEC sprouting assay and in accordance with the findings of Weng et al. (2012), the treatment of murine aortic rings with 6-shogaol strongly and significantly reduced the VEGF-triggered number of sprouts as well as the total sprout length per aortic ring. Moreover and of strong importance, 6-shogaol was able to interfere with already existing angiogenic sprouts, and this effect was recovered after further cultivation of the aortic rings without additional treatment with 6-shogaol. To the best of our knowledge, this is the first report demonstrating the beneficial effect of 6-shogaol for controlled vascular disruption. We suggest that the inhibitory effects of 6-shogaol on VEGF-induced angiogenic sprouting might be, at least in part, attributed to the significant reduction in the VEGFR2 level.

Although the *in vivo* oral application of 6-shogaol is known to result in low plasma levels of the ginger constituent, several approaches are under investigation to improve the bioavailability of 6-shogaol (Asami et al., 2010; Wang et al., 2018; Zhang et al., 2019). Therefore, and due to our findings, 6-shogaol can be suggested as a beneficial substance for a pharmacotherapeutical approach to treat inflammation-related diseases. Since our study was conducted only in *in vitro* and *ex vivo* models, further studies are needed to confirm our results in more physiologically relevant systems. However, we have tried to simulate physiologically relevant situations, for example, by performing a flow-based cell adhesion assay mimicking the physiological blood flow. Although *in vitro* assays lack the involvement of several cell types, we made an attempt to analyze the effects of 6-shogaol exclusively on endothelial cells. Future studies should be performed in advanced models combining different cell types being relevant for angiogenesis- and inflammation-related processes. Nevertheless, by the performance of an *ex vivo* aortic ring assay, we made an attempt to mimic the physiological situation with several cell types that represent a more complex but well-balanced regulation of angiogenic sprouting.

Taken together, the data from our study characterize the main bioactive ingredient in dried ginger, 6-shogaol, as an inhibitor of inflammation- and angiogenesis-related processes in vascular endothelial cells. These findings warrant further exploitation of this ginger compound preclinically.

## Data Availability

The original contributions presented in the study are included in the article/Supplementary Material; further inquiries can be directed to the corresponding author.
