# Supplementary material for: Ginger Constituent 6-Shogaol Inhibits Inflammation- and Angiogenesis-Related Cell Functions in Primary Human Endothelial Cells
Source: Front Pharmacol. 2022 Feb 25;13:844767. doi: 10.3389/fphar.2022.844767 (PMC8914105; doi:10.3389/fphar.2022.844767)
Supplement: Supplementary file 1 [file Presentation1.pdf]

Supplementary information

Supplementary Materials and Methods

Lactate dehydrogenase (LDH) assay

The measurement of LDH in cell culture supernatants after 6-shogaol treatment was performed according to the manufacturer's instructions (CytoTox96®Non-Radioactive Cytotoxicity Assay, Promega, Heidelberg, Germany). For this, confluent HUVECs with a density of 100 % were treated with the indicated concentrations of 6-shogaol or vehicle (DMSO 0.03%) for 24, 48 and 72 h. 45 minutes before the end of each stimulation period, lysis buffer was added to control cells. Subsequently, a substrate solution was added to cell culture supernatants, and, after 30 min of incubation at room temperature, the enzymatic reaction was halted by the addition of stopping solution. Absorption was measured at 490 nm in a plate reader (VarioskanFlash, Thermo Fisher Scientific, Dreieich, Germany).

Cell viability assay

Confluent HUVECs with a density of 100 % were exposed to the indicated concentrations of 6-shogoal over a period of 24, 48 and 72 h and 6-gingerol over a period of 24 and 48 h. 4 h before the end of the incubation time, CellTiter-Blue reagent (Promega, Heidelberg, Germany) was added to the cells. Subsequently, conversion of resazurin to fluorescent resorufin was measured at 535 nm (ex) and 590 nm (em) using a microplate reader (SPECTRAFluor Plus; Tecan, Männedorf, Switzerland).

Cell adhesion assay

Confluent HUVECs with a density of 100 % were treated with indicated concentrations of 6-shogaol for 30 min, before the cells were induced with LPS (1 µg/ml). 24 h later, Jurkat cells were fluorescence-labeled with CellTracker Green (Thermo Fisher Scientific, Schwerte, Germany) and 100,000 cells per well were allowed to adhere onto a 6-shogoal-treated and LPS-activated HUVEC monolayer. Non-adherent Jurkat cells were removed, and adherent cells were measured at 485 nm (ex) and 535 (em) using a plate reader (SPECTRAFluor Plus, Tecan, Männedorf, Switzerland).

Flow cytometry

Confluent HUVECs with a density of 100 % were treated with indicated concentrations of 6-shogaol. 30 min later, the cells were activated with IL-1β (5 ng/ml) or TNF (10 ng/ml) for 4 h (E-selectin) or 24 h (ICAM-1, VCAM-1), before they were stained for ICAM-1 (FITC-coupled, mouse, anti-human CD54, 1:33, MCA1615F; Bio-Rad, Hercules, CA, USA), VCAM-1 (PE-coupled, mouse, anti-human CD106, 1:20, 555647; BD Biosciences, San Jose, CA, USA) or E-selectin (PE-coupled, mouse, anti-human CD62E, 1:10, 551145; BD Biosciences, San Jose, CA, USA). Levels of the respective surface protein were determined using flow cytometry (FACSVerse; BD Biosciences, San Jose, CA, USA).

Western blot analysis

Confluent HUVECs with a density of 100 % were treated with the indicated concentrations of 6-shogaol for 30 min, before they were activated with IL-1β (5 ng/ml) or TNF (10 ng/ml) for 4 h (E-selectin) or 24 h (ICAM-1, VCAM-1). For the analysis on JNK and p38 activation, confluent HUVECs were treated with 6-shogoal (30 µM) for 24 h before MAPK phosphorylation was induced by LPS (1 µg/ml) for 1 and 2 h, respectively. Subsequently, the cells were lysed using RIPA buffer containing protease and phosphatase inhibitors. The total protein in each sample was determined using a Pierce BCA Protein Assay Kit (Thermo Fisher Scientific), and a pyronin-based sample buffer containing sodium dodecyl sulfate (SDS) was added before they were incubated at 95°C for 5 min for denaturation. 35 µg of each sample was subjected to a discontinuous SDS-polyacrylamide gel electrophoresis (PAGE) for protein separation before they were transferred onto a polyvinylidene fluoride membrane (PVDF; Bio-Rad Laboratories, Munich, Germany) by electroblotting for 1 h at 100 V. For blocking of unspecific binding sites, the membranes were incubated for 2 h in 5 % Blotto (Carl Roth, Karlsruhe, Germany) or 5 % bovine serum albumin (BSA; MilliporeSigma, Burlington, MA, USA) containing 0.1 % Tween-20 (Sigma-Aldrich; St. Louis, MO, USA). The membranes were incubated with respective antibodies for detection of the protein of interest: anti-ICAM-1 (rabbit, anti-human, 1:1,000 in 5 % Blotto + Tween-20, 4°C overnight, #4915; Cell Signaling/New England Biolabs, Frankfurt am Main, Germany), anti-VCAM-1 (mouse, anti-human, 1:1,000 in 5 % Blotto + Tween-20, 4°C overnight, sc-13160; Santa Cruz Biotechnology, Dallas, TX, USA), anti-E-selectin (mouse, anti-human, 1:2,000 in 5 % Blotto + Tween-20, 4°C overnight, sc-137054; Santa Cruz Biotechnology, Dallas, TX, USA), anti-SAPK/JNK (rabbit, anti-human, 1:1,000 in 5 % BSA + Tween-20, 4°C overnight, #9258, Cell Signaling/New England Biolabs, Frankfurt am Main, Germany), anti-phospho-SAPK/JNK (Thr183/Tyr185) (rabbit, anti-human, 1:2,000 in 5 % Blotto + Tween-20, 4°C overnight, #9255, Cell Signaling/New England Biolabs, Frankfurt am Main, Germany), anti-p38MAPK (rabbit, anti-human, 1:1,000 in 5 % BSA + Tween-20, 4°C overnight, #9212, Cell Signaling/New England Biolabs, Frankfurt am Main, Germany) and anti-phospho-p38MAPK (Thr180/Tyr182) (rabbit, anti-human, 1:1,000 in 5 % BSA + Tween-20, 4°C overnight, #4511, Cell Signaling/New England Biolabs, Frankfurt am Main, Germany). Anti-β-actin-peroxidase (mouse, anti-human, 1:50,000 in 1 % BSA + Tween-20, 1 h room temperature, A3854; Sigma-Aldrich, St. Louis, MO, USA) was used to detect β-actin as loading control. Secondary antibodies were applied to membranes being incubated with non-conjugated primary antibodies: anti-rabbit (goat, 1:2,000 in 5 % Blotto + Tween-20, 2 h room temperature, #7074; Cell Signaling/New England Biolabs, Frankfurt am Main, Germany) and anti-mouse, HRP-linked antibody (horse, 1:2,000 in 5 % Blotto + Tween-20, 2 h room temperature, #7076; Cell Signaling/New England Biolabs, Frankfurt am Main, Germany). Protein detection was achieved by chemiluminescence measurement, and quantification of specific protein levels in each sample was performed by densitometric analysis using ImageJ (software version 1.49k).

Quantitative polymerase chain reaction (qPCR)

Confluent HUVECs with a density of 100 % were treated with indicated concentrations of 6-shogoal for 30 min before they were activated with IL-1β (5 ng/ml) or TNF (10 ng/ml) for 2 h (E-selectin) or 10 h (ICAM-1, VCAM-1). RNA was isolated using the RNeasy mini kit (Qiagen, Hilden, Germany) according to the manufacturer's instructions, and 1 µg was transcribed into cDNA using the SuperScript reverse transcriptase (Life Technologies, Darmstadt, Germany). The $2^{-\Delta\Delta Ct}$ method was used for quantitative real-time PCR data analysis. GAPDH served as housekeeping gene and the

following primers were used. The following primers were used: E-selectin (forward: 5’-AGA-TGA-GGA-CTG-CGT-GGA-GA-3‘; reverse: 5’-GTG-GCC-ACT-GCA-GGA-TGT-AT-3’), VCAM-1 (forward: 5’-CCA-CAG-TAA-GGC-AGG-CTG-TAA-3’; reverse: 5’-GCT-GGA-ACA-GGT-CAT-GGT-CA-3’), ICAM-1 (forward: 5’-CTG-CTC-GGG-GCT-CTG-TTC-3’; reverse: 5’-AAC-AAC-TTG-GGC-TGG-TCA-CA-3’), IL-6 (forward: 5’-GGT-ACA-ATC-CTC-GAC-GGC-ATC-T-3’; reverse: 5’-GTG-CCT-CTT-TGC-TGC-TTT-CAC-3’); IL-8 (forward: 5’-TGG-CAG-CCT-TCC-TGA-TTT-CT-3’; reverse: 5’-TTA-GCA-CTC-CTT-GGC-AAA-ACT-G-3‘); CXCL12 (forward: 5’-GAA-AGC-CAT-GTT-GCC-AGA-GC-3‘; reverse: 5’-AGC-TTC-GGG-TCA-ATG-CAC-A-3‘), IL-10 (forward: 5’-AGG-CAT-GCA-CAG-CTC-AGC-ACT-GCT-3‘; reverse: 5’-CTG-CTC-CAC-GGC-CTT-GCT-CTT-GTT-3’) and GAPDH (forward: 5’-CCA-CAT-CGC-TCA-GAC-ACC-AT-3’; reverse: 5’-TGA-AGG-GGT-CAT-TGA-TGG-CAA-3’).

Supplementary figure legend

Supplementary Figure 1: Chemical structures of 6-shogoal (A) and 6-gingerol (B). The dashed line indicates the Michael acceptor moiety.

Supplementary Figure 2: Treatment with 6-shogaol up to 30 µM over a period up to 72 h does not impair cell viability in HUVECs. Confluent HUVECs were treated with indicated concentrations of 6-shogaol for 24, 48 and 72 h. (A) To determine compound-derived impact on LDH release and, therefore, impaired membrane integrity, the cell culture supernatants were incubated with a substrate solution, before LDH leakage was measured at 490 nm using a plate reader. The incubation of a lysis solution (45 min) served as positive control. (B) Mitochondrial activity was assessed by the addition of CellTiter-Blue reagent 4 h before the endpoint of compound incubation. Cell viability was determined using a plate reader. Data are expressed as mean ± SD; (A) n=3; *p ≤ 0.05 vs ctrl; (B) n=3; *p ≤ 0.05 vs ctrl.

Supplementary Figure 3: 6-Shogoal reduced the adhesion of Jurkat cells onto LPS-induced HUVECs. Confluent HUVECs were treated with rising concentrations of 6-shogaol, before the cells were inflammatorily activated with LPS (1 µg/ml) for 24 h. Fluorescence-labeled Jurkat cells were allowed to adhere for 5 min, before non-adherent cells were removed. The amount of adherent cells was determined by fluorescence measurement at 485 nm (ex) and 535 nm (em) using a plate reader. Data are expressed as mean ± SD; n=3; *p ≤ 0.05 vs LPS ctrl.

Supplementary Figure 4: TNF-induced CAMs are attenuated by 6-shogaol but less pronounced than in LPS-activated HUVECs. Confluent HUVECs were treated with indicated concentrations of 6-shogoal for 30 min before they were activated by TNF (10 ng/ml) for (A, B) 4 h (E-selectin) and 24 h (ICAM-1, VCAM-1) or for (C) 2 h (E-selectin) and 12 h (ICAM-1, VCAM-1). (A) CAMs on the HUVEC surface were determined by flow cytometry and (B) total protein levels were assessed by western blot analysis. Actin served as loading control. One representative blot is shown. (C) mRNA results were obtained by qPCR. GAPDH served as housekeeping gene. 6-SG: 6-shogaol. Data are expressed as mean ± SD; n=3; *p ≤ 0.05 vs TNF.

Supplementary Figure 5: 6-Shogaol reduces IL-1β-induced CAMs in HUVECs. Confluent HUVECs were treated with indicated concentrations of 6-shogaol for 30 min, before CAM induction was provoked by IL-1β (5 ng/ml) for (A, B) 4 h (E-selectin) and 24 h (ICAM-1, VCAM-1) or for (C) 2 h (E-selectin) and 12 h (ICAM-1, VCAM-1). (A) Levels of CAMs on the surface of HUVECs were

assessed by flow cytometry, and (B) total protein for ICAM-1, VCAM-1 and E-selectin were determined by western blotting. Actin served as loading control. One representative blot is shown. (C) mRNA expression was analyzed by qPCR. GAPDH served as housekeeping gene. 6-SG: 6-shogaol. Data are expressed as mean ± SD; (A-C) n=3; (B) n=5/5/4; *p ≤ 0.05 vs IL-1β.

Supplementary Figure 6: 6-Shogoal markedly attenuates pro-inflammatory cytokines (mRNA) and JNK activation (protein), while IL-10 and p38 remained unimpaired. (A-C) Confluent HUVECs were treated with indicated concentrations of 6-shogoal for 30 min before LPS (1 µg/ml) was added for 10 h. mRNA analysis of IL-6, IL-8, CXCL12 and IL-10 was performed by qPCR. GAPDH served as housekeeping gene. (D) Effects on JNK and p38 phosphorylation were obtained by western blot analysis. Confluent HUVECs were treated with 6-shogaol (30 µM) for 24 h, before the phosphorylation was induced with LPS (1 µg/ml) for 1 or 2 h. Actin served as loading control. One representative blot is shown. 6-SG: 6-shogaol. Data are expressed as mean ± SD; (A-C) n=3; *p ≤ 0.05 vs LPS; (D) n=3.

Supplementary Figure 1

A

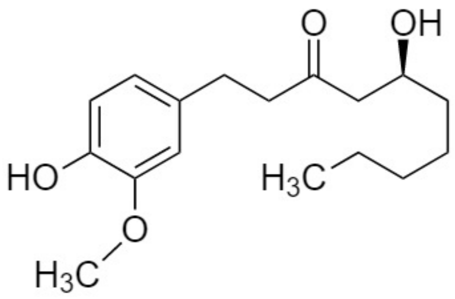

6-gingerol

B

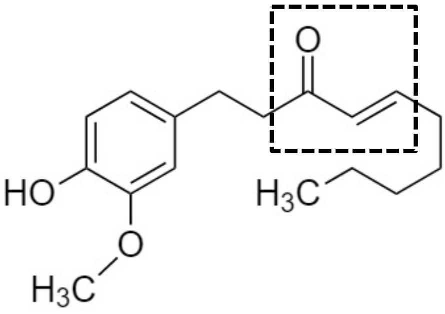

6-shogaol

Supplementary Figure 1: Chemical structures of 6-gingerol (A) and 6-shogaol (B). The dashed line indicates the Michael acceptor moiety.

Supplementary Figure 2

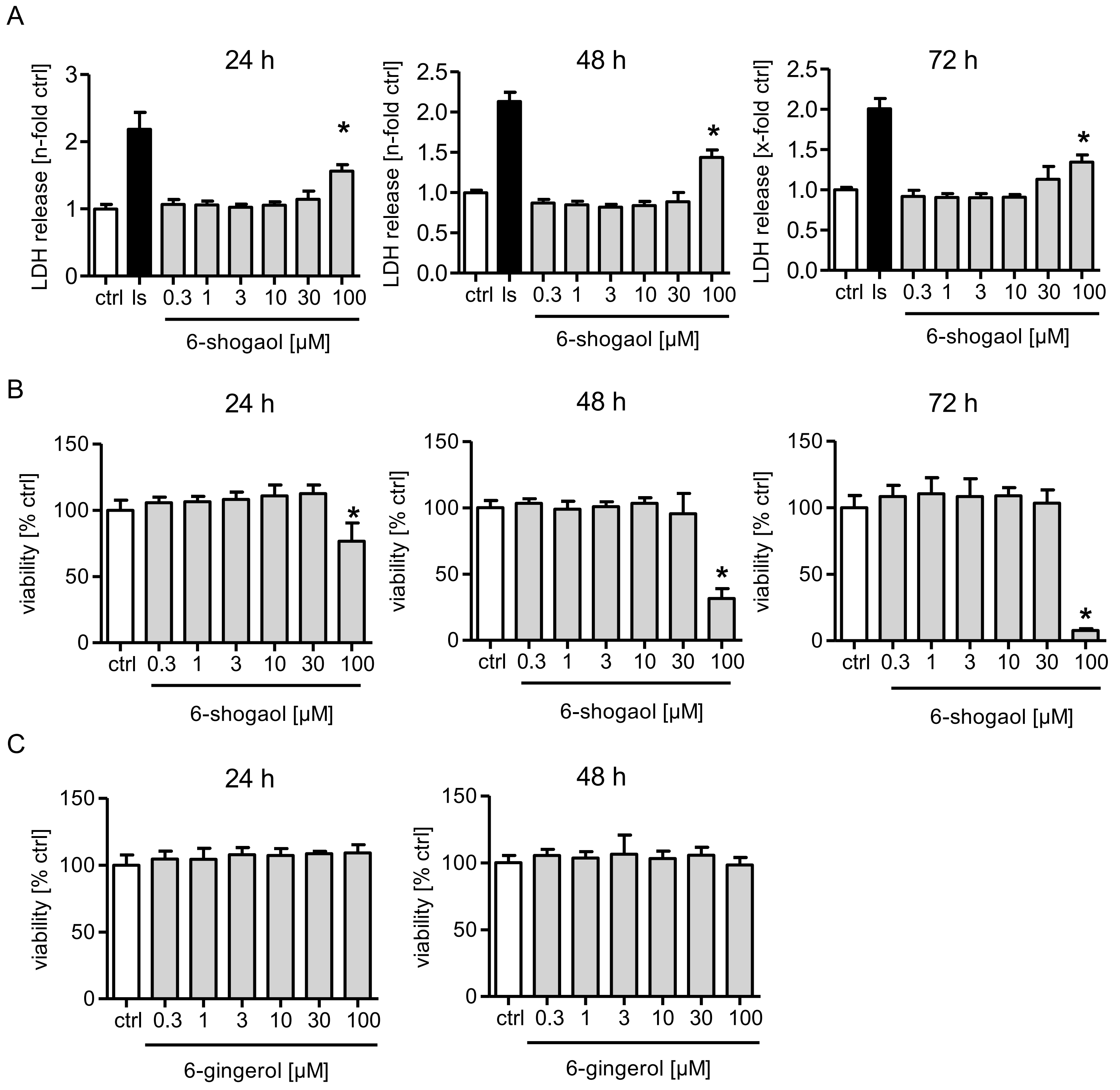

Supplementary Figure 2: Treatment with 6-shogaol up to 30 µM over a period up to 72 h does not impair cell viability in HUVECs. Confluent HUVECs were treated with indicated concentrations of 6-shogaol for 24, 48 and 72 h. (A) To determine compound-derived impact on LDH release and, therefore, impaired membrane integrity, the cell culture supernatants were incubated with a substrate solution, before LDH leakage was measured at 490 nm using a plate reader. The incubation of a lysis solution (45 min) served as positive control. (B) Mitochondrial activity was assessed by the addition of CellTiter-Blue reagent 4 h before the endpoint of compound incubation. Cell viability was determined using a plate reader. (C) Confluent HUVECs were treated with indicated concentrations of 6-gingerol for 24 and 48 h. Mitochondrial activity was assessed by the addtion of CellTiter-Blue reagent 4h before the endpoint of compound incubation. Data are expressed as mean ± SD; (A-C) n=3; *$p \leq 0.05$ vs ctrl.

Supplementary Figure 3

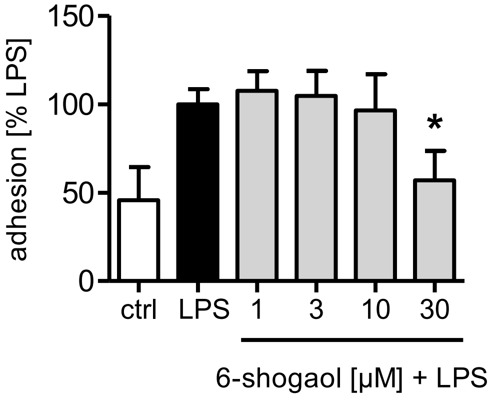

Supplementary Figure 3: 6-Shogaol reduced the adhesion of Jurkat cells onto LPS-induced HUVECs. Confluent HUVECs were treated with rising concentrations of 6-shogaol, before the cells were inflammatorily activated with LPS (1 µg/ml) for 24 h. Fluorescence-labeled Jurkat cells were allowed to adhere for 5 min, before non-adherent cells were removed. The amount of adherent cells was determined by fluorescence measurement at 485 nm (ex) and 535 nm (em) using a plate reader. Data are expressed as mean ± SD; n=3; *$p \leq 0.05$ vs LPS ctrl.

Supplementary Figure 4

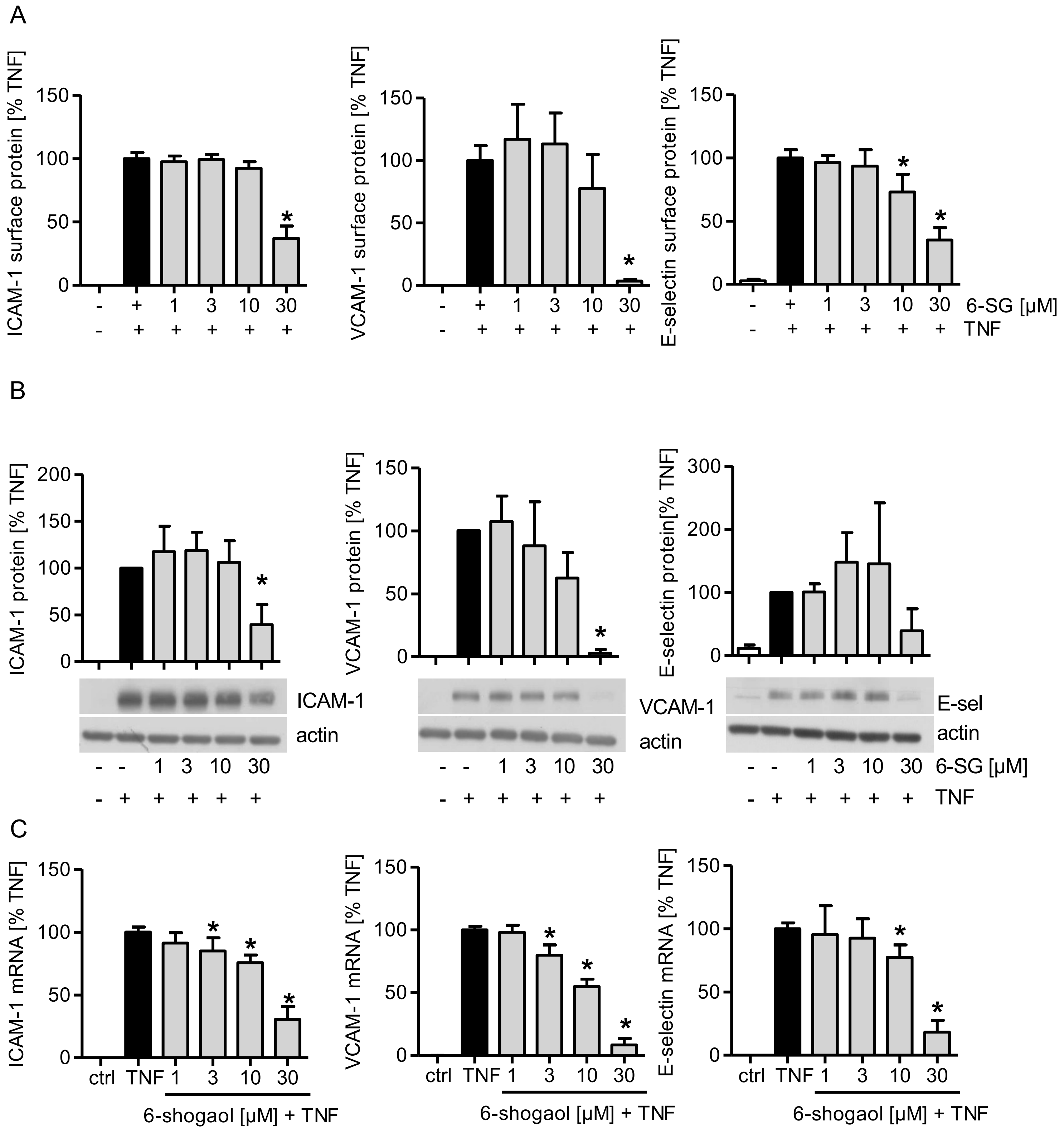

Supplementary Figure 4: TNF-induced CAMs are attenuated by 6-shogaol but less pronounced than in LPS-activated HUVECs. Confluent HUVECs were treated with indicated concentrations of 6-shogaol for 30 min before they were activated by TNF (10 ng/ml) for (A, B) 4 h (E-selectin) and 24 h (ICAM-1, VCAM-1) or for (C) 2 h (E-selectin) and 12 h (ICAM-1, VCAM-1). (A) CAMs on the HUVEC surface were determined by flow cytometry and (B) total protein levels were assessed by Western blot analysis. Actin served as loading control. One representative blot is shown. (C) mRNA results were obtained by qPCR. GAPDH served as housekeeping gene. 6-SG: 6-shogaol. Data are expressed as mean ± SD; n=3; *p ≤ 0.05 vs TNF.

Supplementary Figure 5

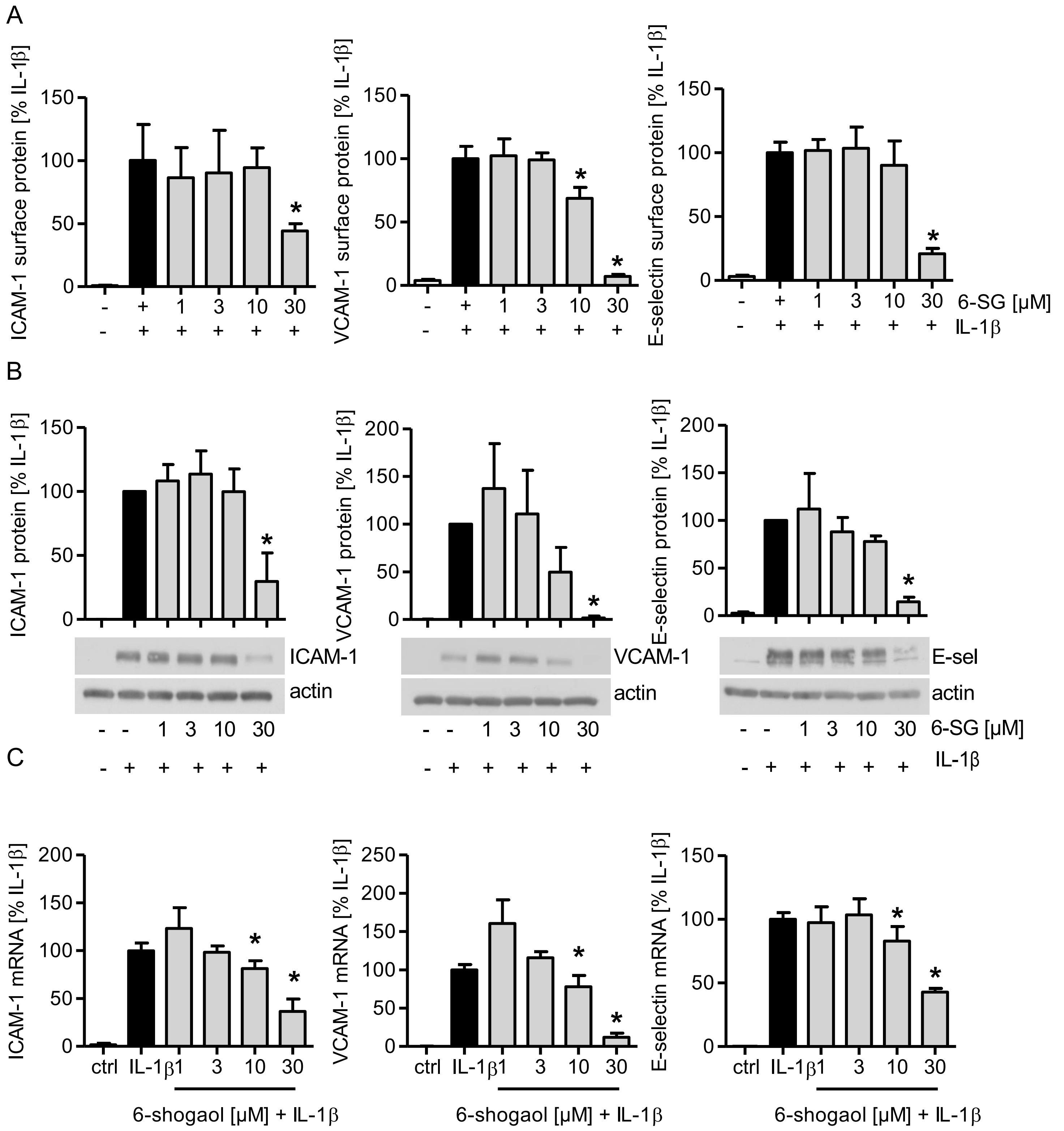

Supplementary Figure 5: 6-Shogaol reduces IL-1β-induced CAMs in HUVECs. Confluent HUVECs were treated with indicated concentrations of 6-shogaol for 30 min, before CAM induction was provoked by IL-1β (5 ng/ml) for (A, B) 4 h (E-selectin) and 24 h (ICAM-1, VCAM-1) or for (C) 2 h (E-selectin) and 12 h (ICAM-1, VCAM-1). (A) Levels of CAMs on the surface of HUVECs were assessed by flow cytometry, and (B) total protein for ICAM-1, VCAM-1 and E-selectin were determined by Western blotting. Actin served as loading control. One representative blot is shown. (C) mRNA expression was analyzed by qPCR. GAPDH served as housekeeping gene. 6-SG: 6-shogaol. Data are expressed as mean ± SD; (A-C) n=3; (B) n=5/5/4; *$p \leq 0.05$ vs IL-1β.

Supplementary Figure 6

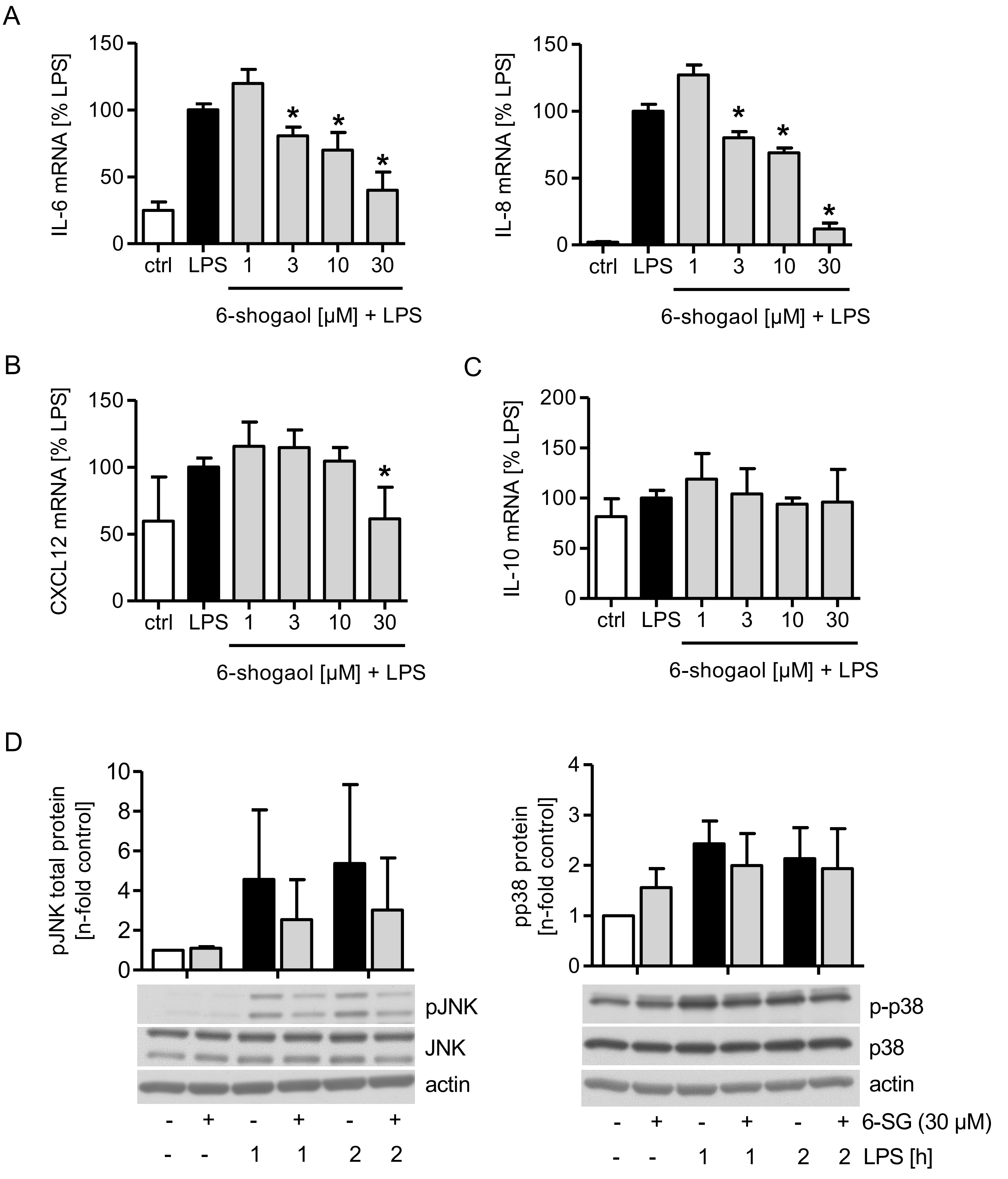

Supplementary Figure 6: 6-Shogaol markedly attenuates pro-inflammatory cytokines (mRNA) and JNK activation (protein), while IL-10 and p38 remained unimpaired. (A-C) Confluent HUVECs were treated with indicated concentrations of 6-shogaol for 30 min before LPS (1 µg/ml) was added for 10 h. mRNA analysis of IL-6, IL-8, CXCL12 and IL-10 was performed by qPCR. GAPDH served as housekeeping gene. (D) Effects on JNK and p38 phosphorylation were obtained by Western blot analysis. Confluent HUVECs were treated with 6-shogaol (30 µM) for 24 h, before the phosphorylation was induced with LPS (1 µg/ml) for 1 or 2 h. Actin served as loading control. One representative blot is shown. 6-SG: 6-shogaol. Data are expressed as mean ± SD; (A-C) n=3; *p ≤ 0.05 vs LPS; (D) n=3.
